# Hippocampal subfields: volume, neuropathological vulnerability and cognitive decline in Alzheimer’s and Parkinson’s disease

**DOI:** 10.1186/s13195-025-01768-w

**Published:** 2025-05-30

**Authors:** Maud M. A. Bouwman, Irene Frigerio, Chen-Pei Lin, Niels Reijner, Wilma D. J. van de Berg, Laura E. Jonkman

**Affiliations:** 1 Department of Anatomy and Neurosciences, Section Clinical Neuroanatomy and Biobanking, Amsterdam UMC, location VUmc, De Boelelaan 1118, Amsterdam, The Netherlands; 2 Amsterdam Neuroscience, Neurodegeneration, Amsterdam, The Netherlands; 3 Amsterdam Neuroscience, Brain imaging, Amsterdam, The Netherlands

**Keywords:** Hippocampal subfields, Post-mortem MRI, MRI volumes, Neuropathology, Hippocampal atrophy, Alzheimer’s disease, Parkinson’s disease

## Abstract

**Background:**

The hippocampus is highly affected in neurodegenerative diseases, including Alzheimer’s disease (AD) and Parkinson’s disease (PD). The relationship between neuropathology and atrophy in hippocampal subfields is complex due to differences in the selective neuronal vulnerability to distinct protein aggregates that underlie cognitive impairment. The aim of the current study was to investigate the relation between hippocampal subfield volumes, neuropathological burden (amyloid-β, p-tau and α-synuclein) and cognitive performance in AD, PD and control brain donors, using a cross-disease and within-subject post-mortem in situ MRI and neuropathology approach.

**Methods:**

A total of 60 brain donors, including 14 non-neurological controls, 27 AD and 19 PD, underwent post-mortem in situ MRI. From 3D-T1 images hippocampal subfield and entorhinal cortex volumes were derived using FreeSurfer-based subfield segmentation. Hippocampal tissue was obtained at subsequent autopsy, fixed and immunostained for amyloid-β, p-tau and pSer129-αSyn. Immunoreactivity in hippocampal subfields was quantified as area% load using QuPath. Clinical Dementia Rating scores were extracted from the clinical files when available.

**Results:**

AD showed atrophy and increased p-tau, but not amyloid-β, burden in the CA1, subiculum and entorhinal cortex compared to controls, however MRI and neuropathology did not correlate. Controls and PD had similar hippocampal subfield volumes and pathology load. In PD, p-tau pathology, rather than pSer129-αSyn, was associated with lower total hippocampal volume (*r*=-0.68, *p* = 0.045), predominantly in PD with dementia (PDD) (*r*=-0.99, *p* = 0.013). Cross-disease, volume loss of the subiculum (*r=-*0.68, *p =* 0.001) and entorhinal cortex (*r=-*0.73, *p =* 0.004) strongly associated with cognitive impairment. Moreover, p-tau pathology had the strongest effect on subfield atrophy, most pronounced in the subiculum (*β=-0.570*, *p* < 0.001), but could only explain 22–44% of the volumetric variance.

**Conclusions:**

Even though p-tau was the strongest predictor of hippocampal subfield atrophy, AD-pathology (p-tau and amyloid-β) only partially accounted for volumetric differences in hippocampal subfields, highlighting the significance of other pathologies or mechanisms. The increased sensitivity of subicular and entorhinal cortical atrophy compared to total hippocampal atrophy highlights the potential clinical value of incorporating hippocampal subfield atrophy in monitoring disease progression.

**Supplementary Information:**

The online version contains supplementary material available at 10.1186/s13195-025-01768-w.

## Background

The hippocampus is a complex and heterogeneous cortical brain structure consisting of multiple interconnected subfields, each with distinct histological characteristics, functions and implications in the formation and retrieval of memory [1, 2]. The hippocampus is highly affected in neurodegenerative diseases, including Alzheimer’s disease (AD) and the progressive stages of Parkinson’s disease (PD).

Hippocampal atrophy is one of the most well-established and widely used MRI biomarkers to assess the degree of hippocampal atrophy in AD and PD with dementia (PDD) [3, 4, 5]. While most neuroimaging studies have evaluated the hippocampus as a singular structure [6, 7, 8], structural changes within the hippocampal subfields reflect differential subfield vulnerability [9, 10]. The specific atrophy pattern seen in AD shows atrophy of the entorhinal cortex followed by the progressive involvement of the cornu Ammonis (CA) 1 and subiculum [2, 9, 11, 12], while in PD the CA2-3, CA4 and the dentate gyrus (DG) are most prominently affected [13]. It is believed that these volumetric changes result from neurodegeneration induced by protein aggregation.

The hippocampus is highly vulnerable to protein aggregations. According to the Braak staging system for neurofibrillary tangles in AD, the transentorhinal region of the medial temporal lobe (MTL) is the first to be affected by tau pathology, before spreading to the entorhinal cortex and the connected hippocampal formation, followed by adjacent association cortices [14, 15]. While phosphorylated tau (p-tau) begins to accumulate in the MTL, amyloid-β deposition starts in the neocortex before spreading to the entorhinal region and CA1, and eventually all interconnected hippocampal subfields [16]. These AD-related pathologies are thought to synergize, disrupting the functional integrity of neural circuits, affecting cognitive functioning [17, 18, 19, 20, 21]. In turn, PD is characterized by the presence of α-synuclein-containing Lewy bodies (LB) and Lewy neurites (LN) [22]. Aggregations of α-synuclein in the limbic system and neocortex are linked to the development of PDD [23], with some studies suggesting LNs in the CA2 region may serve as an initial trigger of cognitive decline [24, 25, 26]. Furthermore, post-mortem studies have reported that around half of the PD cases have additional AD co-pathology[27, 28, 29], associated with cognitive dysfunctioning [30, 31]. The presence of both cortical PD and AD neuropathology was shown to be a better neuropathological correlate of PDD than any of the pathologies in isolation [32].

Although atrophy patterns show concordance with histopathological vulnerability patterns in AD and PD [10, 25, 33, 34], the relationship between neuropathology and atrophy in hippocampal subfields is complex due to differences in the selective neuronal vulnerability to distinct protein aggregates that underlie cognitive dysfunction. Various studies have correlated both semi-quantitative neuropathology scores and quantitative neuropathological load to ex vivo 7T MRI of formalin fixed brains [35, 36, 37]. These studies show an association between tau pathology and atrophy of the CA1, subiculum and entorhinal cortex in various neurodegenerative diseases, including AD and PD. However, translating these results back to the clinic is challenging due to the ex vivo formalin fixed nature of the brain specimens. To mitigate this, a study used ante-mortem 1.5T MRI and post-mortem neuropathological validation [38]. They found a relationship between higher p-tau burden and CA1 and subiculum deformation [38]. However, the time between MRI scans and death was highly variable, up to almost 5 years, which makes the association with progressing neuropathology questionable.

The aim of the current study was to investigate the relation between neuropathological burden (amyloid-β, p-tau and α-synuclein) and hippocampal subfield volumes in AD and PD and age-matched control brain donors. We used post-mortem in situ (brain in cranium) 3T MRI as a proxy for ante-mortem MRI, and correlated volumetric data with quantitative neuropathological burden at the same time point. This approach enables more precise pathological verification while closely reflecting the MRI used in the clinical (research) setting. The results of this study contribute to a better understanding of the underlying pathological profiles that give rise to selective hippocampal subfield atrophy across neurodegenerative diseases.

## Methods

### Donor inclusion

**Fig. 1:**
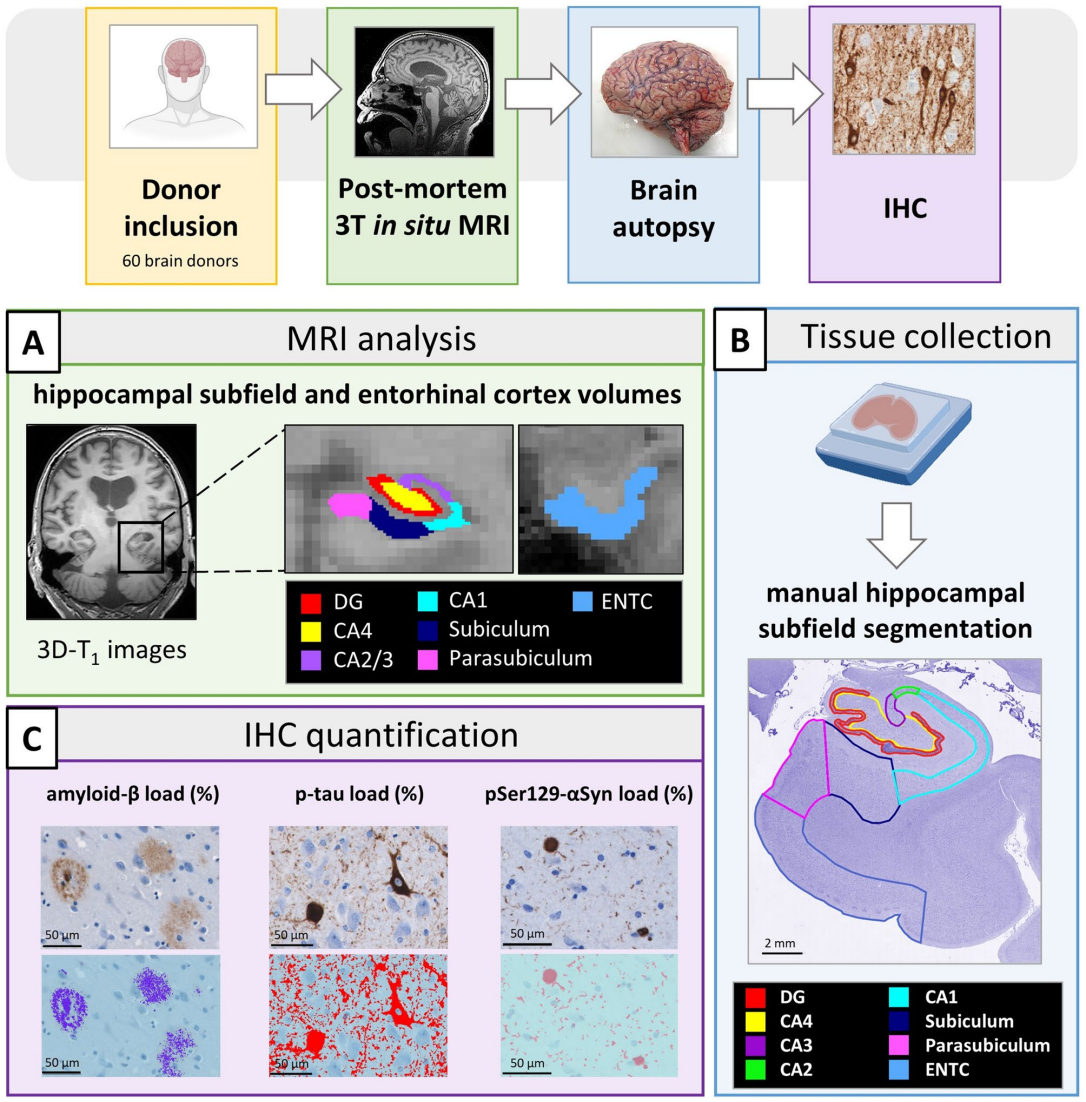
Workflow of the study. Upon donor inclusion (yellow box), post-mortem in situ MR 3D-T1 images were collected for FreeSurfer-based hippocampal subfield [50] and entorhinal cortex segmentation, from which volume in mm^3^ was derived (**A**). Directly after the in situ 3T MRI scan, brain autopsy was performed and the hippocampus medialis was selected from the right hemisphere (blue box). The hippocampus tissue was processed for immunohistochemistry targeting amyloid-β (clone 4G8), p-tau (clone AT8) and phosphorylated Ser129 α-synuclein (pSer129-αSyn, clone EP1536Y), and subsequently imaged using a whole-slide scanner (Vectra Polaris, objective 20x) (purple box). Manual segmentation of the hippocampal subfields (see details in Suppl. Figure 1) was performed in QuPath using the haematoxylin counterstain channel and based on cytoarchitecture as previously described by Adler et al. [54] delineating the DG, CA1-4, subiculum, parasubiculum and entorhinal cortex (**B**). Subsequently, pixel classifiers were used to quantify amyloid-β, p-tau and pSer129-αSyn load (%) in QuPath (**C**). **Legend**: CA: cornu ammonis; DG: dentate gyrus; ENTC: entorhinal cortex; IHC: immunohistochemistry; pSer129-αSyn: phosphorylated Ser129 α-synuclein; p-tau: phosphorylated tau

A total of 60 brain donors, including 14 non-neurological controls, 27 AD and 19 PD, were included in this study. Within the AD group, several clinical phenotypes could be distinguished: amnestic (i.e. typical AD, *N* = 14) and non-amnestic (i.e. atypical AD, *N* = 13)^39^, consisting of behavioral/dysexecutive (B/D, *N* = 5), logopenic variant primary progressive aphasia (lvPPA, *N* = 3), posterior cortical atrophy (PCA, *N* = 4) and for 1 donor the clinical phenotype could not be defined. PD donors could be subdivided based on the presence of dementia (PD (*N* = 10) and PDD (*N* = 9)). The donors were selected from the Normal Aging Brain Collection Amsterdam (NABCA; http://nabca.eu) [40] and the Netherlands Brain Bank (NBB; http://brainbank.nl). The control donors had no reported history of neurological illnesses. The clinical diagnosis was neuropathologically confirmed by an expert neuropathologist according to the international guidelines of the Brain Net Europe II (BNE) consortium (https://www.brainbank.nl/about-us/brain-net-europe/). From neuropathological diagnosis, Thal phase [16], Braak NFT [41] and Braak LB [22] stages, as well as the presence of cerebral amyloid angiopathy (CAA) [42] and limbic-predominant age-related TDP-43 encephalopathy (LATE) [43] were collected. The following inclusion criteria from the neuropathological report were considered for controls: Thal phase ≤ 2 and Braak NFT stage ≤ 1. AD donors were included based on the AD neuropathologic change score of ‘intermediate’ or ‘high’ [44]. In addition, for both controls and AD donors, Braak LB stage > 2 was used as an exclusion criterion, to avoid LB pathology in the hippocampus. All clinically defined PD donors with Braak LB stage > 0 were included. Age at diagnosis, disease duration (age at death minus age at diagnosis) and Clinical Dementia Rating (CDR) scores [45] as measure for overall cognition, were collected from the clinical files and reported in Supplementary Table 1 when available. All donors signed a written informed consent for brain donation and the use of their brain tissue and medical records for research purposes. Upon inclusion, the donors underwent post-mortem in situ MRI and brain autopsy. The workflow is summarized in Fig. 1.

### MRI acquisition and analysis of hippocampal subfields and entorhinal cortex volume

Post-mortem 3T in situ MR images were acquired according to a previously described pipeline [40]. Briefly, MRI scans were acquired with a 3T Signa-MR750 system (General Electric Medical Systems, United States) with an eight-channel phased-array head-coil including (i) a sagittal 3D T1-weighted fast spoiled gradient echo (GRE) sequence [repetition time (TR) = 7 ms, echo time (TE) = 3 ms, flip angle = 15°, 1-mm-thick axial slices, in-plane resolution = 1.0 × 1.0 mm^2^]; and (ii) a sagittal 3D fluid attenuation inversion recovery (FLAIR) sequence [TR = 8000 ms, TE = 130 ms, inversion time (TI) = 2000–2500 ms, 1.2-mm-thick axial slices, in-plane resolution = 1.11 × 1.11 mm^2^]. The TI was optimized for each individual case to account for differences in CSF suppression due to body temperature. The FLAIR images were used for scoring white matter lesions according to the Fazekas grading score [46].

The 3D-T_1_ images were lesion-filled [47] to minimize the impact of age-related white matter abnormalities (e.g. vascular change) on automated segmentation, as previously described [48]. Image processing was performed using FreeSurfer (FS) image analysis version 6.0 (https://surfer.nmr.mgh.harvard.edu/). The Desikan-Killiany atlas [49] was used for total hippocampus and entorhinal cortex parcellation and volume extracting. In addition, intracranial volume (ICV) was obtained. Hippocampal subfield segmentation was performed on both hemispheres according to the integrated hippocampal subfield atlas by Iglesias et al. [50], identifying the following hippocampal subfields with the use of the FS60 protocol: dentate gyrus (DG), CA4, CA3 (including the CA2), CA1, subiculum and parasubiculum (including the presubiculum). Visual inspection of segmentation quality was performed after segmentation. MRI volumetric data from the left hemisphere was not used for further correlation analysis due to the unavailability of corresponding neuropathological data. Normalized whole-brain volume was estimated from 3D-T_1_ images using SIENAX [51], FMRIB Software Library (FSL) tools version 5.0.9 (https://fsl.fmrib.ox.ac.uk/fsl/). An expert neuroradiologist reported additional scoring for medial temporal lobe atrophy (MTA) and white matter lesions (Fazekas).

### Hippocampus tissue sampling and immunohistochemistry

MRI acquisition was followed by brain autopsy. The right hemisphere was fixed in 4% formalin for 4 weeks. Subsequently tissue from the hippocampus medialis, including the entorhinal cortex, was collected for paraffin embedding, and 6-µm–thick sections were cut for immunohistochemical analysis. Hippocampal sections were stained for amyloid-β (clone 4G8), p-tau (clone AT8) and phosphorylated Ser129 α-synuclein (pSer129-αSyn, clone EP1536Y), as previously described by Frigerio et al. [52] The AD cohort was not stained for pSer129-αSyn, as cases were selected on Braak LB stage [22]  ≤ 1, which by definition excludes the presence of αSyn in the hippocampus. Primary antibody details are provided in Supplementary Table 2. Briefly, the sections were deparaffinized and rehydrated using graded alcohol series, followed by antigen retrieval in a steam cooker for 30 minutes. The sections were blocked in 3% normal donkey serum in tris buffered saline (TBS; Triton 0.5%) and incubated in primary antibodies, diluted in 1% normal donkey serum in TBS (Triton 0.1%), overnight at 4°C. After washing in TBS, primary antibodies were detected using EnVision (Dako, Glostrup, Denmark), and visualized using 3.3’-Diaminobenzidine (DAB, Dako) with Imidazole in Tris–HCl (pH 7.6). Haematoxylin was used as counterstain after which the sections were dehydrated in graded alcohol series, treated with xylene and mounted with Entellan (Merck, Darmstadt, Germany).

### Hippocampus segmentation and image analysis

**Fig. 2:**
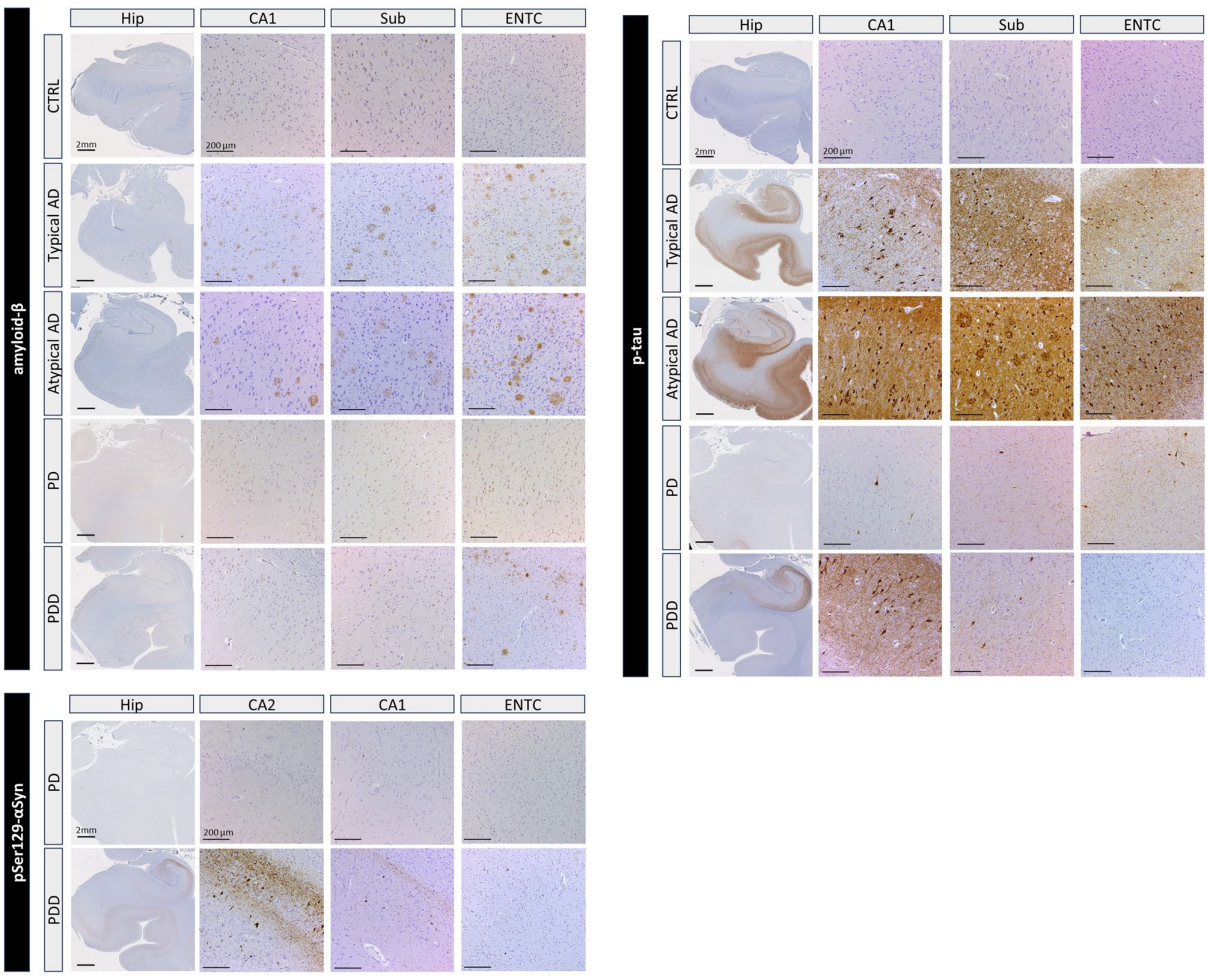
Overview of pathological stainings (amyloid-β, p-tau and pSer129-αSyn) for the total hippocampus and subfields per clinical subgroup. The following antibodies were used: 4G8 for amyloid-β, AT8 for p-tau and EP1536Y for pSer129-αSyn. The scale bar for the hippocampus (Hip) is 2 mm and for the subfields (CA1, CA2, Sub and ENTC) 200 μm. **Legend**: AD: Alzheimer’s disease; CA: cornu ammonis; CTRL: controls; ENTC: entorhinal cortex; Hip: total hippocampus; PD: Parkinson’s disease; PDD: Parkinson’s disease dementia; p-tau: phosphorylated tau; Sub: subiculum

Images of the immunostained hippocampal sections were taken using a whole-slide scanner (Vectra Polaris, 20x objective) and quantified using QuPath 0.2.3 stardist (https://qupath.readthedocs.io/en/0.2/index.html) [53]. Hippocampal sections were manually segmented into individual subfields in the haematoxylin channel, blinded to the neuropathology, based on cytoarchitectural boundaries as previously described in Adler et al. [54]. The hippocampal subfields segmentation included the DG, CA4, CA3, CA2, CA1, subiculum, parasubiculum and entorhinal cortex to match the MRI-derived subfields. The cellular morphological features used to delineate the subfields are explained in Supplementary Fig. 1. DAB immunoreactivity was quantified with previously published *in-house* QuPath scripts [48, 52]. Pixel classifiers were used to measure the %area load for amyloid-β, p-tau and pSer129-αSyn for each hippocampal subregion. An example of the different pathological stainings in different hippocampal subfields is depicted in Fig. 2.

### Statistical analysis

Statistical analysis was performed using IBM SPSS version 28.0 (Chicago, IL). All variables were tested for normality, and demographics between all three groups were compared using a one-way ANOVA for continuous data, and a Chi-square test for categorical variables, e.g. pathological staging. To compare hippocampal MRI volumes between controls and AD or PD, an ANCOVA was used correcting for age at death, sex, post-mortem delay and ICV. The volumetric differences between groups, presented as percentages in the figures, were calculated using estimated marginal means to correct for covariates. Histopathological differences were analyzed with the use of ANCOVA with age at death and sex as covariates. For the subfield analyses, p-values were corrected with false discovery rate (FDR) [55] to correct for multiple comparisons for the different regions included. When > 2 groups (i.e. control, AD, PD) were analyzed, FDR-correction was applied for multiple groups (for total hippocampus) or different regions (for hippocampal subfields). Partial correlations were used to analyze within-group associations between MRI volumes and histopathological measures or CDR scores, correcting for age at death, sex, post-mortem delay and ICV. Only the MRI volumes of the right hemisphere were used for association with histopathological measures, due to the availability of only ipsilateral hippocampal tissue. FDR correction was applied for all regions, including the total hippocampus. A linear regression model with a backward elimination was used for the combined AD and PD group to analyze which predictors (amyloid-β, p-tau, age at death, and ICV) had the strongest effect on the hippocampal subfield volume across disease groups. ICV was included as a covariate in all models while the other predictors were sequentially removed based on statistical significance until best model fit was reached. Control cases were excluded from this analysis due to their limited pathological load, and pSer129-αSyn load was excluded as predictor, as the AD cohort was α-synuclein negative. Prediction models were not run on the clinical groups separately, due to the small sample size. P-values after FDR-correction lower than 0.05 were considered significant.

### Data availability

The data that support the findings of this study are available from the corresponding author upon reasonable request. QuPath scripts used for pathological quantification are openly available at our GitHub repository: https://github.com/NeuroScaleLab/pathological_quantification.

## Results

### Cohort description

Clinical, radiological and neuropathological data of all groups are summarized in Table 1. For detailed information per donor, see Supplementary Table 1. Sex, age at diagnosis and post-mortem delay did not differ between groups. PD donors had a significantly higher disease duration than AD (*p* < 0.001) and were older at death compared to controls (*p* = 0.024). Only 29% of the controls were carrier of one APOE ε4 allele, while almost 60% of the AD group had at least one APOE ε4 allele. AD showed lower normalized brain volume on MRI compared to controls and PD (*p* < 0.001 and *p* = 0.031, respectively). Hippocampal volume of AD donors was lower than controls and PD donors (*p* = 0.001 and *p* = 0.011, respectively). Moreover, AD and PD had higher MTA scores compared to controls (*p* = 0.002 and *p* = 0.004, respectively). Fazekas score, indicative for white matter hyperintensities, was lower in AD and higher in PD compared to controls (*p* = 0.033 and *p* = 0.003, respectively). By definition, AD donors showed significantly higher Thal phase and Braak NFT stage than controls (both *p* < 0.001), while PD donors had a higher Braak NFT and Braak LB stages compared to controls (both *p* < 0.001). None of the control donors had LATE, and the presence of LATE was not different between disease groups (*p* = 0.716). Furthermore, AD donors exhibited more cerebral amyloid angiopathy (CAA) compared to both controls and PD (*p* < 0.001).

**Table 1:** Donor characteristics

	Control	AD	PD
**Clinical characteristics**			
N	14	27	19
**Sex** F/M (% F)	8/6 (57%)	8/19 (30%)	8/11 (42%)
**Age at diagnosis** years, mean [range]	/	61 [35–82]	63 [44–84]
**Disease duration** years, mean [range]	/	5 [0–13]	16 [8–23]***
**Age at death** years, mean [range]	70 [57–85]	67 [37–84]	79 [62–94]^#^
**Post-mortem delay** hours: min, mean [range]	9:06 [6:50 − 12:45]	7:46 [3:35 − 11:30]	7:50 [3:30 − 10:40]
**CDR** median (N) [range]	N/A	3.0 (*N* = 17) [1–3]	1.5 (*N* = 9) [0.5-3]
**APOE genotype** N (%) ε4 non-carrier ε4 heterozygous ε4 homozygous	14 10 (71%) 4 (29%) 0	24 10 (42%) 12 (50%) 2 (8%)	N/A
**Radiological characteristics**			
**NBV** liters, mean ± SD	1.46 ± 0.07	1.34 ± 0.09^###^	1.40 ± 0.07*
**Hippocampal volume** mm^3^, mean ± SD	3375 ± 314	2871 ± 516^##^	3217 ± 401*
**MTA score** median [range]	0 [0–2]	2 [0–4]^##^	1.25 [1–4]^##^
**Fazekas score** median [range]	1.5 [0–2]	1 [0–3]^#^	2 [0–3]^##^
**Pathological characteristics**			
**Thal phase** N 0/1/2/3/4/5	3/7/4/0/0/0	0/0/0/1/2/24^###^	1/7/4/5/2/0
**Braak NFT stage** N 0/1/2/3/4/5/6	4/10/0/0/0/0/0	0/0/0/0/3/7/17^###^	0/3/9/4/3/0/0^###^
**Braak LB stage** N 0/1/2/3/4/5/6	12/2/0/0/0/0/0	26/1/0/0/0/0/0	0/0/0/0/1/1/17^###^
**CAA** N absent/type 1/type 2	11/2/1	1/17/9^###^	12/2/5***
**LATE** N (%) stage 0/1/2/3	0 (0%) 14/0/0/0	6 (22%) 21/3/1/2	3 (16%) 16/0/3/0

**Legend**: *AD: Alzheimer’s disease; CAA: cerebral amyloid angiopathy; CDR: clinical dementia rating; F: female; LB: Lewy body; M: male; MTA: medial temporal lobe atrophy; N/A: not available*,* NBV: normalized brain volume; NFT: neurofibrillary tangles; PD: Parkinson’s disease; SD: standard deviation.* * *p* < 0.05, ** *p* < 0.01, *** *p* < 0.001 compared to AD; ^#^*p* < 0.05, ^##^*p* < 0.01, ^###^*p* < 0.001 compared to control

### Controls show only minimal pathology in the hippocampus

In aged control brain donors (mean [range]: 70 [57–85] years) overall hippocampal MRI volume was 3375 mm^3^ (SD: ± 314 mm^3^). The highest hippocampal subfield volume was observed in the CA1 (629 ± 69 mm^3^), followed by the subiculum (421 ± 45 mm^3^), parasubiculum (361 ± 45 mm^3^), DG (282 ± 36 mm^3^), CA4 (240 ± 32 mm^3^) and CA2/3 (216 ± 29 mm^3^) (Suppl. Figure 2A). In addition, the entorhinal cortex was 1756 mm^3^ (± 219 mm^3^). There was only minimal pathology in the hippocampus of the control group. Amyloid-β load seemed equally distributed across hippocampal subfields (mean [range]: 0.8% [0.04–2.6%]) (Suppl. Figure 2B), mostly aggregated as diffuse plaques, while p-tau burden seemed slightly higher in the CA1 (0.9%), subiculum (0.9%), parasubiculum (0.7%) and entorhinal cortex (0.9%) compared to other regions, reflecting mostly neurofibrillary threads (NTs) and some neurofibrillary (pre-)tangles (NFTs) (Suppl. Figure 2C). Within controls, no correlations were found between MRI hippocampal subfield volumes and pathology load (Suppl. Figure 2D & Suppl. Table 3).

### Selective vulnerability of the CA1, subiculum and entorhinal cortex in AD

**Fig. 3:**
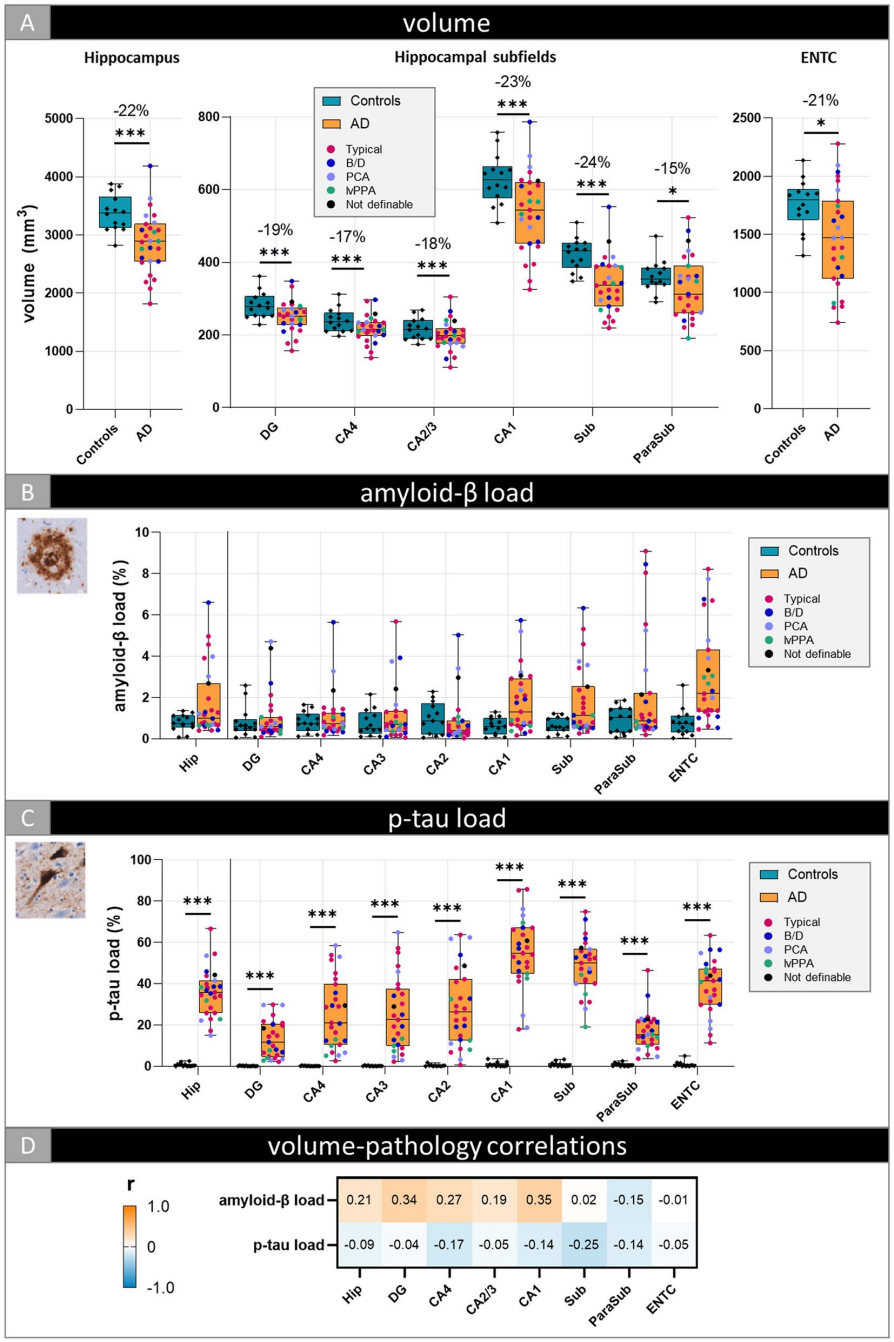
Differences in hippocampal subfield volume and pathology load between controls and AD. Volumetric (**A**), amyloid-β (**B**) and p-tau load (**C**) differences between controls and AD are shown for the total hippocampus and per hippocampal subregion, and correlations between volume and pathology load within the AD group (**D**). Every data point represents one donor, and for the AD cohort color-coded based on clinical phenotype. The boxes indicate the median with 25th and 75th percentile. All p-values were FDR-corrected for multiple comparisons. * *p* < 0.05, ** *p* < 0.01, *** *p* < 0.001. The percentages depicted in (**A**) are the percentage difference in estimated marginal means, taking into account the influence of covariates. The heatmap in (**D**) is color-coded for correlation coefficient (r): blue represents negative and orange positive correlations. **Legend**: AD: Alzheimer’s disease; B/D: behavioral/dysexecutive; CA: Cornu Ammonis; DG: dentate gyrus; ENTC: entorhinal cortex; Hip: total hippocampus; lvPPA; logopenic variant primary progressive aphasia; ParaSub: parasubiculum; PCA: posterior cortical atrophy; Sub: subiculum

AD donors showed a decrease in total hippocampal (-22%, *p* < 0.001) and entorhinal cortical (-21%, *p* = 0.044) MRI volumes compared to controls (Fig. 3A). All hippocampal subfield volumes were significantly lower in AD compared to controls, of which the subiculum showed to be the most atrophic (-24%, *p* < 0.001), followed by CA1 (-23%, *p* < 0.001). Compared to the right hemisphere, left hippocampal subfield volumes seemed to be slightly more atrophic in AD compared to controls, most pronounced in the subiculum (-29%, *p* < 0.001) and entorhinal cortex (-26%, *p* < 0.001) (Suppl. Figure 3). Amyloid-β burden was not statistically different between AD and controls across hippocampal subfields (Fig. 3B). Generally in AD, across all subfields, diffuse plaques were the predominant type of morphological amyloid deposition, with more compact plaques in the subiculum and entorhinal cortex and lake-like amyloid-β depositions in the superficial layers of the parasubiculum and entorhinal cortex. In contrast, p-tau pathology was significantly higher in all AD hippocampal subfields and adjacent entorhinal cortex than in controls (all *p* > 0.001) (Fig. 3C). The CA1 and subiculum showed the highest p-tau burden (55% and 48%, respectively), followed by the entorhinal cortex (39%), all abundant in NFTs and NTs. Less affected subfields (e.g. DG and parasubiculum) showed, descriptively, predominantly NT pathology.

We did not observe any AD-specific correlations between hippocampal subfields volume and amyloid-β or p-tau load (Fig. 3D & Suppl. Table 4).

### AD phenotypes show differences in volume, but not pathological burden

We explored whether the clinical phenotypes within the AD cohort showed hippocampal volumetric differences. When we split the AD group based on the main clinical phenotype (typical vs. atypical), we found that the typical AD phenotype had significant lower CA1 (-15%, *p* = 0.04) and subiculum (-17%, *p* = 0.04) volumes compared to atypical AD (Suppl. Figure 4A). No significant differences in amyloid-β and p-tau burden were observed between typical and atypical AD (Suppl. Figure 4B-C). Descriptively, typical and atypical AD showed similar pathological morphologies. Within the atypical AD group, the variability in hippocampal subfield volumes and pathology load seemed unrelated to clinical subtype. No correlations between volume and pathology load were found within typical and atypical AD phenotypes (Suppl. Figure 4D & Suppl. Table 5).

### Increased p-tau pathology associates with lower total hippocampal volume in PD

**Fig. 4:**
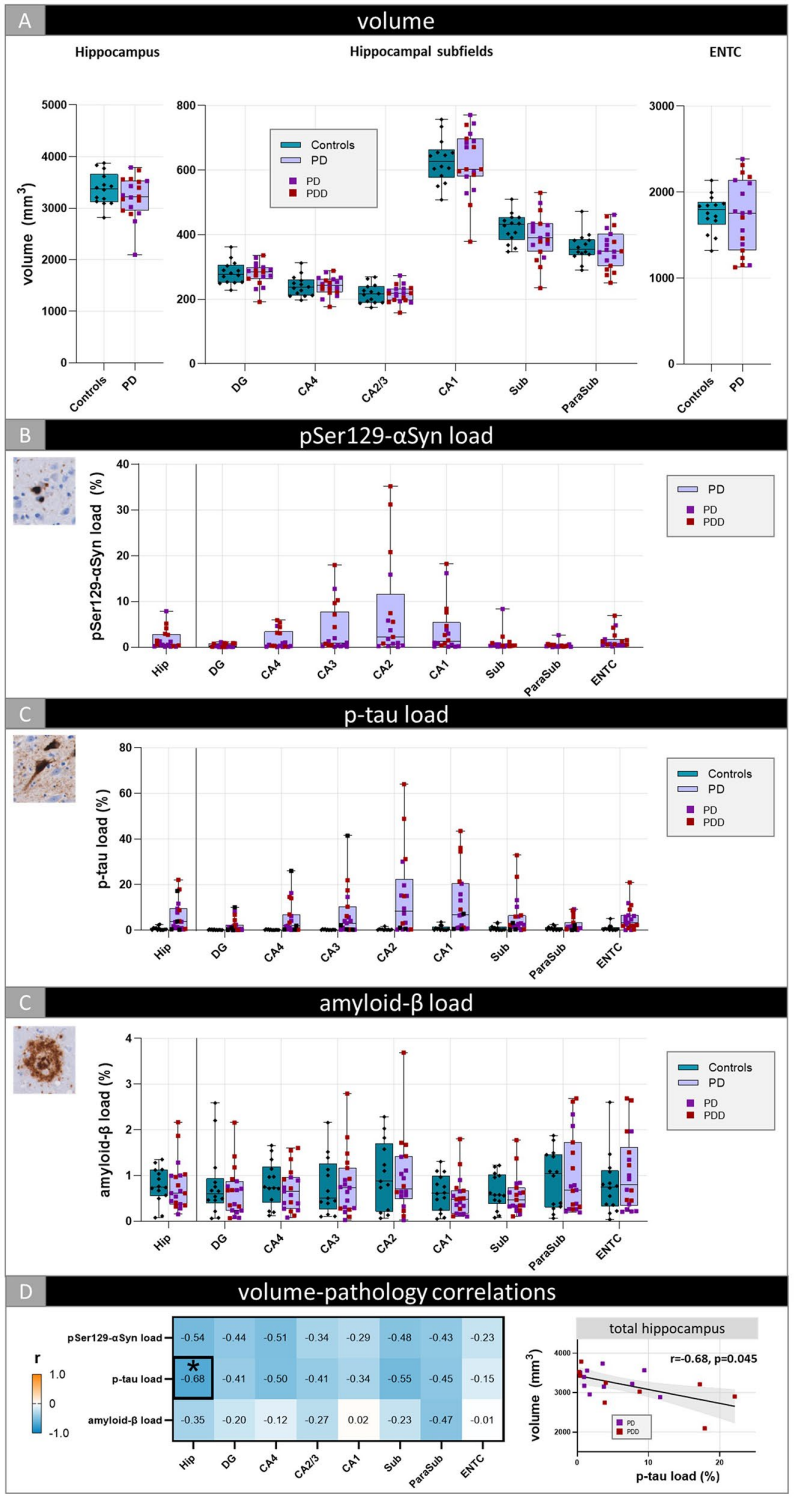
Hippocampal subfield volume and pathology load differences between controls and PD. Volumetric (**A**) and p-tau (**C**) and amyloid-β load (**D**) differences between controls and PD are shown for the total hippocampus and per hippocampal subregion. pSer129-αSyn load (**B**) across hippocampal subfields was shown for PD only, as controls were α-synuclein negative. Correlations between volume and pathology load within the PD group are shown in (**D**). The heatmap is color-coded for correlation coefficient (r): blue represents negative and orange positive correlations. The significant correlation (indicated with an asterisk) between volume and p-tau in the total hippocampus is shown in the scatterplot. Every data point represents one donor and for the PD cohort color-coded based on the presence of dementia (PD vs. PDD). The boxes indicate the median with 25th and 75th percentile. All subfield p-values were FDR-corrected for multiple comparisons. * *p* < 0.05. **Legend**: CA: Cornu Ammonis; DG: dentate gyrus; ENTC: entorhinal cortex; Hip: total hippocampus; ParaSub: parasubiculum; PD: Parkinson’s disease; PDD: Parkinson’s disease dementia; Sub: subiculum

**Fig. 5:**
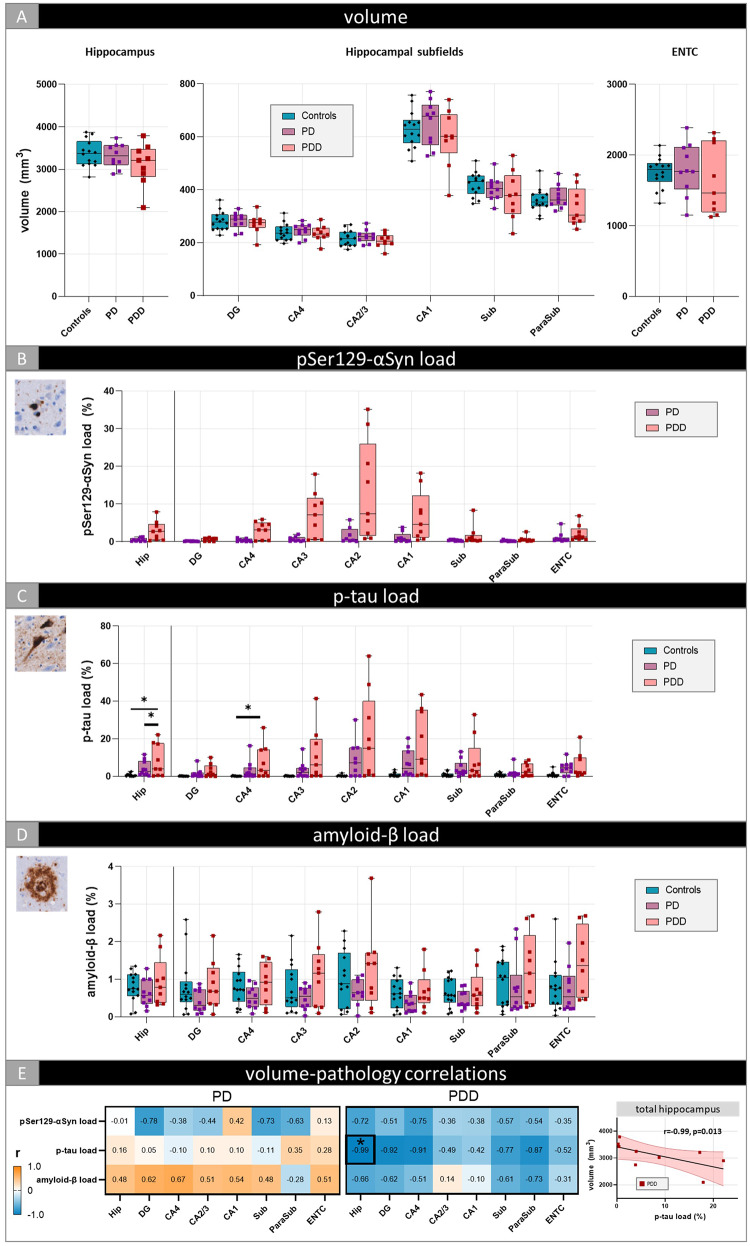
Hippocampal subfield volume and pathology load differences between controls,** PD and PDD.** Volumetric (**A**) and pSer129-αSyn (**B**), p-tau (**C**) and amyloid-β load (**D**) differences between controls and PD donors without (PD) and with dementia (PDD) are shown for the total hippocampus and per hippocampal subregion and correlations between volume and pathology load for the PD and PDD group (**E**). The heatmaps are color-coded for correlation coefficient (r): blue represents negative and orange positive correlations. The significant correlation (indicated with an asterisk) between volume and p-tau in the total hippocampus in PDD is shown in the scatterplot. Every data point represents one donor. The boxes indicate the median with 25th and 75th percentile. All subfield p-values were FDR-corrected for multiple comparisons. * *p* < 0.05. **Legend**: CA: Cornu Ammonis; DG: dentate gyrus; ENTC: entorhinal cortex; Hip: total hippocampus; ParaSub: parasubiculum; PD: Parkinson’s disease; PDD: Parkinson’s disease dementia; Sub: subiculum

Controls and PD donors showed similar MRI volumes across hippocampal subfields (Fig. 4A). When the PD group was split based on the presence of dementia (PD vs. PDD), no significant differences were observed between controls, PD and PDD donors (Fig. 5A). Hippocampal subfield volumes from the left hemisphere did not deviate from the right hemisphere (Suppl. Figure 5).

As per in- and exclusion criteria, control cases were α-synuclein negative and therefore excluded from pSer129-αSyn analysis. At a descriptive level, PD donors showed the highest pSer129-αSyn pathology in the CA2 region, primarily consisting of LNs, although there was a high between-donor variability (Fig. 4B). In PDD donors, substantial pSer129-αSyn pathology was also present in the adjacent CA1 and CA3 region, where it predominantly accumulated in LBs. (Fig. 5B). Furthermore, the CA2-CA1 transition area was densely packed with LNs. While pSer129-αSyn pathology in the CA1, CA2 and CA3 appeared more prominent in PDD than in PD, the difference was not statistically significant (Fig. 5B). Additionally, pSer129-αSyn pathology seemed to predominantly accumulate in LBs within the deeper cortical layers of the entorhinal cortex, regardless of diagnostic classification (PD vs. PDD). Regarding p-tau pathology, PD donors and controls showed similar load across all hippocampal subregions (Fig. 4C). However, split by dementia status, a higher p-tau load was observed in PDD compared to controls, and to PD donors, for the total hippocampus (*p* = 0.024 & *p* = 0.035, respectively) and CA4 (*p* = 0.041) (Fig. 5C). Irrespective of total p-tau pathology burden or diagnostic classification (PD vs. PDD), the presence of both NFTs and NTs seemed to increase with higher levels of p-tau pathology. Amyloid-β burden was similar between PD(D) and controls across hippocampal subregions (Figs. 4C and 5D).

In PD, volume of the total hippocampus decreased with increasing p-tau load (*r*=-0.68, *p* = 0.045) (Fig. 4D & Suppl. Table 6), specifically in PDD donors (*r*=-0.99, *p* = 0.013) (Fig. 5E & Suppl. Table 7). Before FDR-correction, higher pSer129-αSyn load was associated with lower total hippocampal volume in PD (*r*=-0.54, *p* = 0.046), however this finding did not survive multiple comparisons. In the total hippocampus, p-tau and pSer129-αSyn load were highly correlated in PD (*r* = 0.82, *p* < 0.001) (Suppl. Figure 6). This correlation seemed to be specific to PDD (*r* = 0.84, *p* = 0.017), but the latter did not survive multiple comparisons.

### AD and PDD show no hippocampal subfield volume differences, while AD has higher p-tau burden

Both AD and PDD donors presented with cognitive decline (median [range]: 3 [1–3] & 1.5 [0.5-3], respectively). Total hippocampus volume was lower in AD compared to PDD (-12%, *p* = 0.031), but hippocampal subfield volumes did not differ (all *p* > 0.05) (Suppl. Figure 7A). Pathologically, the two groups showed differences (Suppl. Figure 7B&C). In AD p-tau load was higher in the total hippocampus (*p* < 0.001), DG (*p* = 0.049), CA1 (*p* < 0.001), subiculum (*p* < 0.001), parasubiculum (*p* = 0.004) and entorhinal cortex (*p* < 0.001) (Suppl. Figure 7B). The CA2-4 region showed similar p-tau burden in the two groups. No group differences were observed for amyloid-β. The AD group, in contrast to PDD, had no pSer129-αSyn burden due to our selection criteria as described in the methods (Suppl. Figure 7C).

### Subfield volume loss associates with cognition and p-tau pathology cross-disease

**Fig. 6:**
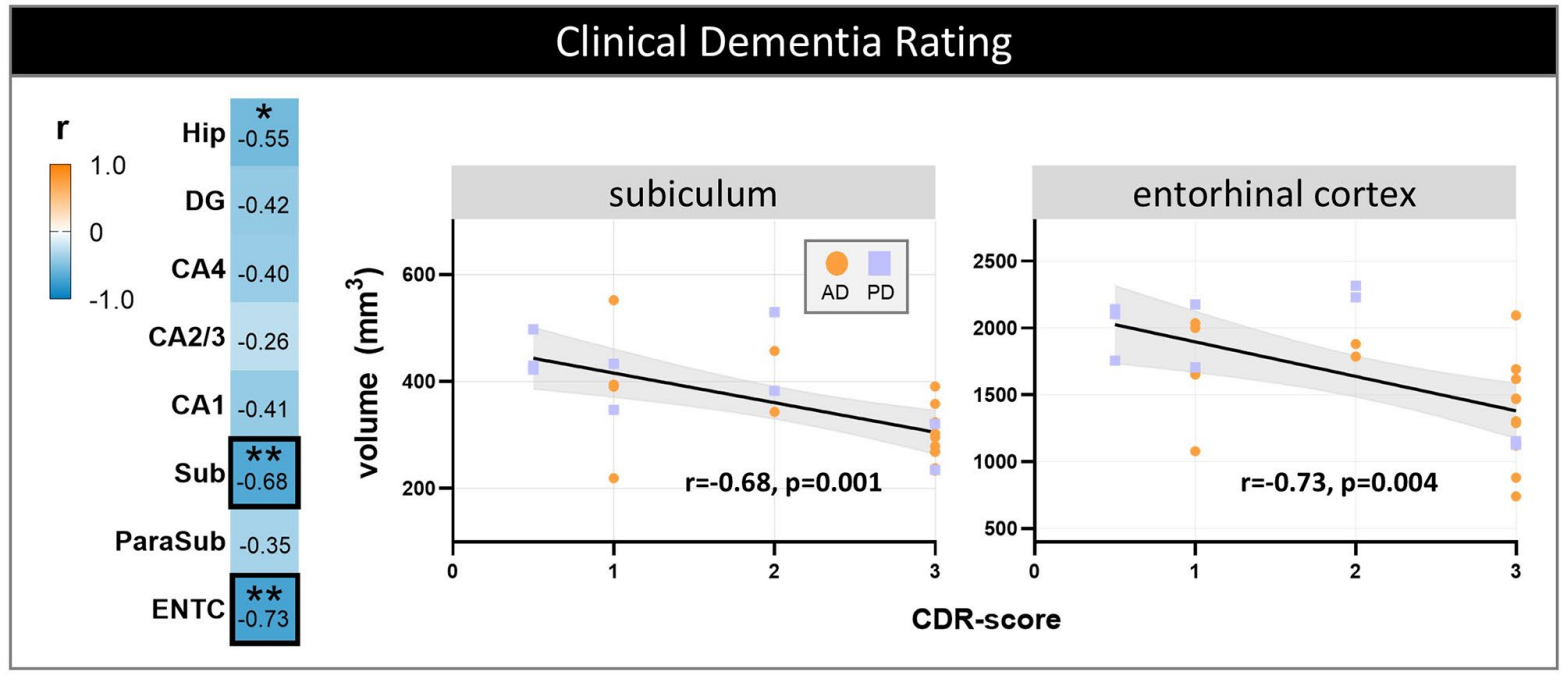
Hippocampal subfield volume associations with cognitive scores. Correlations between volumes and CDR scores are shown per hippocampal (sub)region across disease groups. The heatmap is color-coded for correlation coefficient (r): blue represents negative and orange positive correlations. The correlation coefficient for each hippocampal (sub)region is reported. For the significant correlations the p-values (as asterisks) are also reported and the strongest (with black box) are shown in the scatterplots on the right, color- and shape-coded for group. All p-values were FDR-corrected for multiple comparisons. * *p* < 0.05, ** *p* < 0.01. **Legend**: AD: Alzheimer’s disease; CA: Cornu Ammonis; CDR: clinical dementia rating; DG: dentate gyrus; Hip: total hippocampus; ENTC: entorhinal cortex; ParaSub: parasubiculum; PD: Parkinson’s disease; Sub: subiculum

Global hippocampal volume loss was associated with higher CDR scores (*r*=-0.55, *p* = 0.040), indicating more hippocampal atrophy with greater cognitive impairment. Regionally, a decrease in subiculum and entorhinal cortex volumes were strongly associated with higher CDR scores (*r*=-0.68, *p* = 0.001 and *r*=-0.73, *p* = 0.004, respectively) (Fig. 6). On the contrary, higher MTA score was not correlated with higher CDR scores (*r* = 0.40, *p* = 0.071).

We examined the strongest pathological effect of hippocampal subfield atrophy across disease groups using a linear regression model, independent of clinical diagnosis. Input variables were age at death, ICV and pathological markers amyloid-β and p-tau. Explained variance for MRI volume ranged between 44% (total hippocampus) and 22% (entorhinal cortex). Across most hippocampal subfields, p-tau was the strongest model predictor for hippocampal atrophy (all *p* < 0.035), most pronounced in the subiculum (*β=-0.570*, *p* < 0.001) (Table 2). Only in the CA2/3 and the parasubiculum, p-tau was not a significant contributor to volume decrease. On the contrary, amyloid-β burden significantly contributed to an increase in total hippocampal volume (*β = 0.324*, *p* = 0.024). Regionally, this was only seen in the DG and CA4 (*β = 0.299*, *p* = 0.040 and *β = 0.328*, *p* = 0.018, respectively). Additionally, age at death was only predictive for volume loss in the subiculum (*β=-0.331*, *p* = 0.019). Due to small sample sizes, subgroup analyses were not performed.

**Table 2:** Linear regression models with backwards elimination across disease groups, examining the contribution of pathology (Aβ and p-tau), age at death and intracranial volume to hippocampal subfield volume changes

Region	Strongest model predictors	β-coefficient	*R* ^2^	*p*-value
Hip	Aβ% p-tau% ICV	0.32 -0.50 0.52	0.44	0.024 < 0.001 < 0.001
DG	Aβ% p-tau% ICV	0.30 -0.36 0.58	0.33	0.040 0.017 < 0.001
CA4	Aβ% p-tau% ICV	0.33 -0.42 0.54	0.39	0.018 0.003 < 0.001
CA2/3	ICV	0.53	0.26	< 0.001
CA1	p-tau% ICV	-0.36 0.45	0.30	0.007 < 0.001
Sub	p-tau% Age at death ICV	-0.57 -0.33 0.37	0.41	< 0.001 0.019 0.003
ParaSub	ICV	0.51	0.25	< 0.001
ENTC	p-tau% ICV	-0.29 0.41	0.22	0.035 0.004

Aβ: amyloid-βCA: Cornu Ammonis; DG: dentate gyrus; ENTC: entorhinal cortex; Hip: total hippocampus; ICV: intracranial volume; ParaSub: parasubiculum; R^2^: determination coefficient

## Discussion

In this study, we investigated the contribution of neuropathological burden to hippocampal subfields volume loss in controls, AD and PD brain donors, using an ipsilateral within-subject post-mortem MRI-pathology approach. We found a selective vulnerability of the CA1, subiculum and entorhinal cortex in AD, with no volume-pathology associations. Within an α-synucleinopathy (i.e. PDD), p-tau, rather than pSer129-αSyn load, was associated with hippocampal volume loss. Moreover, atrophy of the subiculum and entorhinal cortex was more sensitive to cognitive decline than total hippocampal volume or a semi-quantitative rating. Finally, irrespective of clinical diagnosis, increased p-tau load was the strongest predictor of atrophy in hippocampal subfields in AD and PD(D).

In controls, hippocampal (subfield) volumes were consistent with those reported in the literature [2, 13, 56, 57]. Some p-tau pathology was observed in the CA1, (para)subiculum and entorhinal cortex, but this did not associate with volume in these regions.

In AD, the CA1, subiculum and entorhinal cortex showed the highest p-tau load and the highest volume loss on MRI. This confirms that early affected regions are selectively vulnerable to neurodegeneration, as postulated by Braak (1991) and others [2, 9, 12, 20, 41, 58, 59, 60]. Previous studies have shown an association between p-tau load and volume loss of hippocampal subfields in AD [12, 33, 35, 38, 37, 61, 62, 63, 64, 65, 66, 67, 68]. In our cohort, we did not find this association. There are several possible explanations for this lack of association in our study. First, it is important to acknowledge a potential selection bias, as the AD cohort may not be representative of the broader AD population due to the younger age at onset and atypical clinical phenotype. Second, volume measurements might lack the sensitivity needed to detect all structural changes in the hippocampal subfields whereas measures of hippocampal shape or (cortical) thickness may be more effective [12, 38, 65]. Third, pathological quantification of one section of the hippocampus medialis might not explain the volumetric atrophy pattern along the longitudinal hippocampal axis [69]. Fourth, we should consider which tau isoform is quantified; the AT8 antibody used in current study only detects pre- and mature tangles, omitting extracellular ghost tangles [70]. In our end-stage AD study, we expect ghost tangles to be present, leading to an underestimation of the total p-tau load. Lastly, a possible explanation is the so-called ceiling effect of tau pathology in AD, in which increases in p-tau load do not correspond with more severe hippocampal volume loss [37, 71]. In support of this hypothesis, our PDD donors showed similar MRI hippocampal subfield volumes compared to AD but had significantly lower p-tau burden which showed an association with total hippocampal atrophy. Moreover, Ravikumar and colleagues [37] found stronger associations between hippocampal subfield thickness and NFT burden in regions affected later by tau pathology, rather than those affected earlier, such as the hippocampus.

We observed total hippocampus, CA1 and subiculum to be more atrophic in typical AD compared to atypical AD, but these regions did not show higher pathological burden in typical AD. Arezoumandan et al. also found no difference in AT8 p-tau load between these two AD phenotypes [72]. However, they did find that a specific truncated tau isoform (E ^391^-truncated tau (MN423)), primarily detecting ghost tangles, was higher in the CA1 and CA2 region of the hippocampus of typical AD. Given that our typical AD may have already reached the most mature state of p-tau pathology in the earliest affected regions [70], this could explain why we found hippocampal subfields to be more atrophic in typical AD. Future research should elucidate whether distinct p-tau isoforms contribute to hippocampal volumetric loss in different AD phenotypes. Additionally, Murray et al. showed that hippocampal sparing AD had larger hippocampal volumes and higher neuronal counts in the CA1-subiculum compared to limbic-predominant cases [73]. Although their stratification was based on pathological distribution (i.e. cortical-to-hippocampal p-tau ratio), hippocampal sparing AD cases often presented with atypical clinical phenotypes, similar to those in our study. In line with this, we observed greater atrophy in total hippocampus, CA1 and subiculum in typical compared to atypical AD. These findings underscore the importance of accounting for clinical heterogeneity when interpreting hippocampal (subfield) volumes in neuroimaging studies.

In our study, we observed similar MRI hippocampal subfields volumes between PD and controls, consistent with some studies [74, 75, 76], while others have reported hippocampal subregional atrophy in the DG, CA4, CA2/3 and subiculum [5, 13, 77, 78]. Additionally, hippocampal (subfield) volumes in PDD were statistically not different from controls or PD without dementia. This is in contrast with previous reports of reduced total hippocampal volume in PDD compared to controls [5, 79] and PD [80, 81, 82] as well as hippocampal subfield atrophy of the CA2-3 and presubiculum in PDD relative to controls [83] and PD [13] Notably, a substantial proportion of these studies [5, 77, 78, 79] used lower-resolution MRI scanners (1.5T) with different and/or older hippocampal subfield segmentation protocols.

It remains unclear whether dementia in PD is primarily associated with α-synuclein pathology, concomitant AD neuropathological changes or a combination of both [84]. In our study, p-tau pathology was more sensitive in discriminating PD cases with and without dementia than pSer129-αSyn pathology. Similarly, Kalaitzakis et al. have shown that AD co-pathology in the entorhinal cortex and CA2 is associated with the presence of dementia in PD [85]. Even though we did not observe hippocampal atrophy in PDD compared to controls or PD, accumulation of p-tau, rather than pSer129-αSyn pathology was associated with a decrease in total hippocampal volume. In the current study, p-tau and pSer129-αSyn load were highly correlated across hippocampal subfields in PDD. This suggests a synergistic interplay that promotes their mutual aggregation [86], potentially involving an enhancing mechanism that may drive neurodegeneration [32, 75, 87, 88, 89, 90]. In addition, Yoshida et al. [71] recently showed that hippocampal accumulation patterns of p-tau, amyloid-β and pSer129-αSyn may be mutually influenced by coexisting pathologies and although the hippocampi of AD donors in the current study lacked α-synuclein pathology, previous research has shown that α-synuclein co-pathology can drive tau accumulation in AD and potentially accelerate the disease phenotype [91].

Despite similar hippocampal subfield volume patterns, AD donors showed a significant increase in p-tau burden across hippocampal subfields compared to PDD, except for the CA2-4 subregions. The distinct pathological accumulation pattern in PDD, in which the CA1, subiculum and entorhinal cortex seem to be less vulnerable to tau aggregation compared to AD, suggests that other mechanisms might be responsible for dementia in PD. For instance, studies have reported α-synuclein pathology in other limbic and neocortical areas to be crucial for the development of PDD [92, 93, 94, 95]. Furthermore, our study exclusively studied pSer129-αSyn pathology which may underestimate the contribution of glial and/or pre-synaptic αSyn to dementia [96].

In CSF, PET and ante- and post-mortem MRI studies, p-tau has been shown to be the strongest predictor of cortical and hippocampal (subfield) atrophy, particularly in the CA1, subiculum and entorhinal cortex [11, 33, 35, 38, 37, 61, 66, 97, 98, 99, 100, 101]. In the current study, the contribution of p-tau load to atrophy varied across subfields, but the subiculum showed a particular vulnerability to p-tau burden. Consistently, a study by Hanko et al. [38] showed hippocampal shape deformation of approximately the CA1/subiculum to be associated with higher NFT burden in AD, even when correcting for other pathologies (amyloid-β and TDP-43). Other studies found similar results, indicating that CA1 and subiculum are the hippocampal subregions where atrophy is most strongly associated with increased p-tau burden [12, 33, 35, 37, 63, 64, 65].

Our prediction models showed a higher amyloid-β load to be predictive of greater hippocampal volume, rather than less. This phenomenon was limited to the total hippocampus and the DG and CA4 subregions. Even though this an unexpected finding in atrophic neurodegenerative disease brains, this has been shown before [99, 102, 103, 104]. In our group, in a subset of AD cases used in present study, Frigerio et al. [48] found a weak positive association between amyloid-β load and global cortical thickness. A possible explanation for this phenomenon could be that amyloid-β load plateaus at demented stages, at which amyloid-β deposition might dissociate from neurodegenerative processes [103, 105]. Another possible explanation is plaque-induced neuroinflammation [106, 107, 108, 109, 110], resulting in local tissue swelling^99^, ^111^ or the space-occupying nature of extracellular amyloid-β plaques, pushing away surrounding tissue and increasing estimates of brain volume [99]. This theory is supported by anti-amyloid clinical trials showing amyloid-β removal leading to cortical thinning while enhancing cognitive performance [112].

Along with age at death and ICV, amyloid-β and p-tau burden could only explain 22–44% of the variability in hippocampal subfield volumes. Other pathologies could also play a major role in the cascade leading up to neurodegeneration. Recently, TDP-43 has been shown to contribute to greater hippocampal atrophy [113], worse disease progression [114, 115, 116, 117, 118], and possibly to worsen AD pathology [117, 119, 120]. Although our donors had limited LATE co-pathology in the hippocampus, quantitative burden of TDP-43 pathology might be insightful and possibly contributing to hippocampal atrophy in the general population. Other pathologies that could attribute to volume loss and disease progression are synaptic dysfunction and loss [121], neuroinflammation [122, 123, 124, hippocampal sclerosis^125^] and vascular pathology [126, 127, 128], but also biological processes such as neuronal shrinkage [129] and autophagy [130].

Atrophy of subiculum and entorhinal cortex seems to be more strongly associated with cognitive decline than total hippocampal atrophy or MTA assessment. Since both the subiculum and entorhinal cortex are important cortico-hippocampal gateway structures [131], atrophy in these regions may contribute to disruption of cortical-hippocampal connections, eventually resulting in cognitive impairment [132, 133]. The subiculum is one of the major output regions of the hippocampus, transmitting hippocampal information to various (sub)cortical areas, and responsible for consolidating memory and memory recall [97]. Given our finding that p-tau strongly contributed to atrophy in the subiculum, we can hypothesize that intracellular p-tau accumulation may disrupt information transmission to the cortex, possibly leading to cognitive decline through neurodegeneration, as has been suggested before [19]. Additionally, the entorhinal cortex, as part of the parahippocampal cortex, acts as a gateway between the cortex and hippocampus innervating particularly the CA1 and CA3, and therefore essential in the formation of episodic memory, memory encoding and retrieval [131]. The entorhinal cortex is one of the first regions to be affected by p-tau and amyloid-β pathology^16^, ^41^, ^134^], potentially disrupting signal transmission from the cortex to the hippocampus and giving rise to cognitive deterioration [132]. Volumetric changes in the subiculum and entorhinal cortex may therefore reflect underlying neuropathology. This supports the idea that neuronal integrity in these subregions is critical for cognitive functioning, and suggests that subfield-specific volumetric assessment could be valuable for monitoring disease progression. To establish this, in vivo studies are needed to examine longitudinal changes in these subregions.

The within-subject MRI and histopathology approach in this study provides translational relevance to the clinical (research) setting. Nevertheless, this approach also has some limitations. Firstly, due to the small sample size, subgroup prediction model analyses were not possible, and therefore the predictive effects of pSer129-αSyn on volumetric changes in PD could not be assessed. Second, comparing our findings to other studies in the research field is complicated due to the large variability in MRI hippocampal segmentations protocols across studies, which currently also lack subfield registration accuracy [9]. Third, the heterogeneity of our relatively small atypical AD cohort might have attenuated potential differences in hippocampal volumes or neuropathological burden [39, 66]. Lastly, it is important to keep in mind that in current study MRI volumes of the whole length of the hippocampus are correlated to pathological quantification of one section of the hippocampus medialis, while it is known that the anterior, medial and posterior portions of the hippocampus can be affected differently by pathology [69]. Future research should investigate MRI-pathology associations in larger cohorts with use of a harmonized hippocampal subfield segmentation protocol [135].

## Conclusions

Using a within-subject post-mortem MRI-pathology approach, p-tau seemed the strongest predictor of hippocampal subfield atrophy in AD and PD(D). AD-pathology (amyloid-β and p-tau) could only account for a part of volumetric changes in hippocampal subfields, highlighting the significance of other pathologies or mechanisms. In PD, concomitant p-tau and its synergistic interplay with α-synuclein pathology, potentially promoting mutual aggregation, appear to contribute to hippocampal atrophy in the demented stages. The increased sensitivity of subicular and entorhinal cortical atrophy compared to total hippocampal atrophy highlights the potential clinical value of incorporating hippocampal subfield atrophy in monitoring disease progression.

## Electronic supplementary material

Below is the link to the electronic supplementary material.

Supplementary Material 1

Supplementary Material 2

## Data Availability

The data that support the finding of this study are available from the corresponding author upon reasonable request. QuPath scripts used for pathological quantification are openly available at our GitHub repository: https://github.com/NeuroScaleLab/pathological_quantification.

## Abbreviations

AD: Alzheimer’s disease
B/D: Behavioral/dysexecutive
CA: Cornu Ammonis
CDR: Clinical Dementia Rating
CSF: Cerebrospinal fluid
DG: Dentate gyrus
ENTC: Entorhinal cortex
ICV: Intracranial volume
IHC: Immunohistochemistry
LB: Lewy body
LN: Lewy neurites
LvPPA: Logopenic variant primary progressive aphasia
MTL: Medial temporal lobe
NFT: Neurofibrillary tangles
NT: Neurofibrillary threads
PCA: Posterior cortical atrophy
PD: Parkinson’s disease
PDD: Parkinson’s disease dementia
p-tau: Phosphorylated tau
pSer129-αSyn: Phosphorylated Ser129 alpha synuclein
