# Supplementary material for: Hippocampal subfields: volume, neuropathological vulnerability and cognitive decline in Alzheimer’s and Parkinson’s disease
Source: Alzheimers Res Ther. 2025 May 30;17:121. doi: 10.1186/s13195-025-01768-w (PMC12124080; doi:10.1186/s13195-025-01768-w)
Supplement: Supplementary file 1 — Supplementary Material 1 [file 13195_2025_1768_MOESM1_ESM.docx]

**Supplementary tables**

**Supplementary Table 1.** Donor characteristics

| Case number | Clinical diagnosis | Sex | Age at diagnosis (years) | Disease duration (years) | CDR | APOE genotype | Age at death (years) | PMD  (hr:min) | Cause of death | ICV (L) | HP volume (mm^3^) | MTA score | Fazekas score | Thal phase^1^ | Braak NFT stage^2^ | Braak LB stage^3^ | ABC score^4^ | CAA type | LATE stage |  |  |  |  |  |  |  |  |  |  |  |  |  |  |  |  |  |  |  |
| --- | --- | --- | --- | --- | --- | --- | --- | --- | --- | --- | --- | --- | --- | --- | --- | --- | --- | --- | --- | --- | --- | --- | --- | --- | --- | --- | --- | --- | --- | --- | --- | --- | --- | --- | --- | --- | --- | --- |
| CONTROLS | | | | | | | | | | | | | | | | | | | |  |  |  |  |  |  |  |  |  |  |  |  |  |  |  |  |  |  |  |
| 1 | CTRL | M | - | - | - | 34 | 68 | 8:30 | Euthanasia | 1.49 | 1.61 | 0.0 | 0.0 | 2 | 1 | 0 | A1B1C0 | 1 | 0 |  |  |  |  |  |  |  |  |  |  |  |  |  |  |  |  |  |  |  |
| 2 | CTRL | F | - | - | - | 23 | 63 | 8:10 | Euthanasia | 1.48 | 1.49 | 0.0 | 0.0 | 0 | 0 | 0 | A0B0C0 | 0 | 0 |  |  |  |  |  |  |  |  |  |  |  |  |  |  |  |  |  |  |  |
| 3 | CTRL | M | - | - | - | 23 | 82 | 10:30 | Liver cirrhosis | 1.57 | 1.39 | 0.0 | 2.0 | 1 | 1 | 0 | A1B1C0 | 0 | 0 |  |  |  |  |  |  |  |  |  |  |  |  |  |  |  |  |  |  |  |
| 4 | CTRL | M | - | - | - | 33 | 85 | 9:22 | Euthanasia | 1.83 | 1.35 | 1.0 | 2.0 | 1 | 1 | 0 | A1B1C0 | 0 | 0 |  |  |  |  |  |  |  |  |  |  |  |  |  |  |  |  |  |  |  |
| 5 | CTRL | F | - | - | - | 33 | 76 | 7:50 | Euthanasia | 1.36 | 1.46 | 1.0 | 2.0 | 2 | 1 | 0 | A1B1C0 | 0 | 0 |  |  |  |  |  |  |  |  |  |  |  |  |  |  |  |  |  |  |  |
| 6 | CTRL | M | - | - | - | 23 | 67 | 8:10 | Liver cirrhosis | 1.45 | 1.43 | 1.0 | 0.0 | 1 | 1 | 0 | A1B1C0 | 1 | 0 |  |  |  |  |  |  |  |  |  |  |  |  |  |  |  |  |  |  |  |
| 7 | CTRL | F | - | - | - | 43 | 57 | 9:50 | Euthanasia | 1.37 | 1.54 | 0.0 | 0.0 | 1 | 0 | 0 | A1B0C0 | 0 | 0 |  |  |  |  |  |  |  |  |  |  |  |  |  |  |  |  |  |  |  |
| 8 | CTRL | F | - | - | - | 33 | 72 | 7:20 | Heart failure | 1.45 | 1.49 | 0.0 | 1.0 | 0 | 0 | 0 | A0B0C0 | 0 | 0 |  |  |  |  |  |  |  |  |  |  |  |  |  |  |  |  |  |  |  |
| 9 | CTRL | F | - | - | - | 33 | 69 | 12:45 | Pulmonary embolism | 1.24 | 1.40 | 1.0 | 2.0 | 1 | 1 | 1 | A1B1C0 | 0 | 0 |  |  |  |  |  |  |  |  |  |  |  |  |  |  |  |  |  |  |  |
| 10 | CTRL | M | - | - | - | 34 | 59 | 8:00 | Euthanasia | 1.50 | 1.45 | 1.0 | 0.0 | 2 | 1 | 0 | A1B1C0 | 0 | 0 |  |  |  |  |  |  |  |  |  |  |  |  |  |  |  |  |  |  |  |
| 11 | CTRL | M | - | - | - | 23 | 77 | 11:25 | Pneumonia | 1.59 | 1.50 | 2.0 | 0.0 | 1 | 1 | 0 | A1B1C0 | 0 | 0 |  |  |  |  |  |  |  |  |  |  |  |  |  |  |  |  |  |  |  |
| 12 | CTRL | F | - | - | - | 33 | 78 | 10:00 | Unknown | 1.39 | 1.50 | 0.0 | 2.0 | 1 | 1 | 1 | A1B1C0 | 0 | 0 |  |  |  |  |  |  |  |  |  |  |  |  |  |  |  |  |  |  |  |
| 13 | CTRL | F | - | - | - | 33 | 59 | 8:10 | Euthanasia | 1.37 | 1.42 | 0.0 | 2.0 | 0 | 0 | 0 | A0B0C0 | 0 | 0 |  |  |  |  |  |  |  |  |  |  |  |  |  |  |  |  |  |  |  |
| 14 | CTRL | F | - | - | - | 34 | 71 | 6:50 | Lung carcinoma | 1.38 | 1.46 | 0.0 | 2.0 | 2 | 1 | 0 | A1B1C0 | 2 | 0 |  |  |  |  |  |  |  |  |  |  |  |  |  |  |  |  |  |  |  |
| AD | | | | | | | | | | | | | | | | | | | |  |  |  |  |  |  |  |  |  |  |  |  |  |  |  |  |  |  |  |
| 15 | Amnestic | M | 60 | 0 | 2 | 33 | 60 | 8:21 | Euthanasia | 1.75 | 1.48 | 0.0 | 3.0 | 5 | 6 | 0 | A3B3C3 | 1 | 0 |  |  |  |  |  |  |  |  |  |  |  |  |  |  |  |  |  |  |  |
| 16 | Amnestic | M | 66 | 2 | 3 | 33 | 68 | 9:09 | Euthanasia | 1.62 | 1.41 | 1.0 | 1.0 | 5 | 5 | 0 | A3B3C3 | 1 | 0 |  |  |  |  |  |  |  |  |  |  |  |  |  |  |  |  |  |  |  |
| 17 | Amnestic | M | 62 | 7 | 3 | 34 | 69 | 11:33 | Pulmonary infection | 1.62 | 1.43 | 3.0 | 0.0 | 5 | 5 | 0 | A3B3C3 | 1 | 0 |  |  |  |  |  |  |  |  |  |  |  |  |  |  |  |  |  |  |  |
| 18 | Amnestic | M | 71 | 13 | 1 | 34 | 84 | 5:53 | Euthanasia | 1.72 | 1.38 | 2.0 | 3.0 | 4 | 5 | 0 | A3B2C2 | 0 | 0 |  |  |  |  |  |  |  |  |  |  |  |  |  |  |  |  |  |  |  |
| 19 | Amnestic | F | 79 | 1 | 1 | 33 | 80 | 7:03 | Epileptic seizure | 1.19 | 1.40 | 2.5 | 1.0 | 5 | 4 | 0 | A3B2C2 | 1 | 1 |  |  |  |  |  |  |  |  |  |  |  |  |  |  |  |  |  |  |  |
| 20 | Amnestic | M | 48 | 5 | NA | 33 | 53 | 9:00 | Dehydration | 2.08 | 1.24 | 1.0 | 0.0 | 5 | 6 | 0 | A3B3C3 | 2 | 0 |  |  |  |  |  |  |  |  |  |  |  |  |  |  |  |  |  |  |  |
| 21 | Amnestic | M | 54 | 10 | 3 | 34 | 64 | 7:55 | Euthanasia | 1.81 | 1.23 | 4.0 | 0.0 | 5 | 6 | 0 | A3B3C3 | 2 | 0 |  |  |  |  |  |  |  |  |  |  |  |  |  |  |  |  |  |  |  |
| 22 | Amnestic | M | 82 | 2 | NA | 33 | 84 | 6:16 | Medication overdose | 1.49 | 1.29 | 2.0 | 2.5 | 3 | 4 | 0 | A2B2C2 | 0 | 0 |  |  |  |  |  |  |  |  |  |  |  |  |  |  |  |  |  |  |  |
| 23 | Amnestic | M | 72 | 5 | NA | 44 | 77 | 6:17 | Euthanasia | 1.39 | 1.34 | 1.5 | 3.0 | 5 | 6 | 0 | A3B3C3 | 2 | 1 |  |  |  |  |  |  |  |  |  |  |  |  |  |  |  |  |  |  |  |
| 24 | Amnestic | M | 62 | 3 | NA | 34 | 65 | 9:18 | Myocardial infarction | 1.64 | 1.44 | 1.0 | 1.0 | 5 | 5 | 0 | A3B3C3 | 1 | 0 |  |  |  |  |  |  |  |  |  |  |  |  |  |  |  |  |  |  |  |
| 25 | Amnestic | M | 53 | 10 | 3 | 43 | 63 | 8:45 | Palliative sedation | 1.60 | 1.27 | 1.5 | 0.0 | 5 | 6 | 0 | A3B3C3 | 2 | 0 |  |  |  |  |  |  |  |  |  |  |  |  |  |  |  |  |  |  |  |
| 26 | Amnestic | F | 51 | 10 | NA | 44 | 61 | 7:40 | End-stage disease | 1.37 | 1.31 | 3.0 | 0.0 | 5 | 6 | 0 | A3B3C3 | 1 | 2 |  |  |  |  |  |  |  |  |  |  |  |  |  |  |  |  |  |  |  |
| 27 | Amnestic | F | 45 | 8 | 3 | 23 | 53 | 6:30 | Euthansia | 1.60 | 1.28 | 2.0 | 3.0 | 5 | 6 | 0 | A3B3C3 | 2 | 0 |  |  |  |  |  |  |  |  |  |  |  |  |  |  |  |  |  |  |  |
| 28 | Amnestic | M | 71 | 8 | NA | 33 | 79 | 8:05 | Unknown | 1.80 | 1.43 | 4.0 | 3.0 | 5 | 6 | 2 | A3B3C3 | 3 | 3 |  |  |  |  |  |  |  |  |  |  |  |  |  |  |  |  |  |  |  |
| 29 | NA | M | 61 | 4 | 2 | 33 | 65 | 7:31 | Cardiac arrest | 1.60 | 1.49 | 1.0 | 2.0 | 4 | 5 | 0 | A3B3C3 | 2 | 0 |  |  |  |  |  |  |  |  |  |  |  |  |  |  |  |  |  |  |  |
| 30 | PCA | M | 57 | 5 | 3 | 34 | 62 | 8:09 | Palliative sedation | 1.54 | 1.18 | 3.5 | 1.0 | 5 | 6 | 0 | A3B3C3 | 1 | 0 |  |  |  |  |  |  |  |  |  |  |  |  |  |  |  |  |  |  |  |
| 31 | B/D | M | 35 | 2 | 1 | 23 | 37 | 11:07 | Euthanasia | 1.89 | 1.46 | 1.0 | 0.0 | 5 | 6 | 0 | A3B3C3 | 3 | 0 |  |  |  |  |  |  |  |  |  |  |  |  |  |  |  |  |  |  |  |
| 32 | B/D | M | 56 | 2 | NA | 43 | 58 | 8:55 | Cachexia | 1.59 | 1.38 | 1.0 | 0.0 | 5 | 6 | 0 | A3B3C3 | 2 | 0 |  |  |  |  |  |  |  |  |  |  |  |  |  |  |  |  |  |  |  |
| 33 | PCA | M | 60 | 7 | 3 | 34 | 67 | 6:21 | Cachexia | 1.68 | 1.27 | 2.0 | 0.0 | 5 | 6 | 0 | A3B3C3 | 1 | 0 |  |  |  |  |  |  |  |  |  |  |  |  |  |  |  |  |  |  |  |
| 34 | B/D | M | 74 | 3 | 1 | 34 | 77 | 7:00 | Euthanasia | 1.55 | 1.51 | 0.5 | 1.0 | 5 | 4 | 0 | A3B2C2 | 1 | 0 |  |  |  |  |  |  |  |  |  |  |  |  |  |  |  |  |  |  |  |
| 35 | B/D | F | 57 | 2 | 3 | 34 | 59 | 3:34 | Dysphagia | 1.64 | 1.30 | 3.0 | 2.0 | 5 | 5 | - | A3B3C3 | 2 | 0 |  |  |  |  |  |  |  |  |  |  |  |  |  |  |  |  |  |  |  |
| 36 | lvPPA | F | 67 | 5 | 3 | 23 | 72 | 5:05 | End-stage disease | 1.32 | 1.23 | 2.0 | 2.0 | 5 | 6 | 0 | A3B3C3 | 1 | 0 |  |  |  |  |  |  |  |  |  |  |  |  |  |  |  |  |  |  |  |
| 37 | B/D | F | 72 | 1 | 3 | 34 | 73 | 6:15 | Pneumothorax | 1.64 | 1.37 | 3.5 | 0.0 | 5 | 6 | 0 | A3B3C3 | 1 | 1 |  |  |  |  |  |  |  |  |  |  |  |  |  |  |  |  |  |  |  |
| 38 | PCA | F | 58 | 2 | NA | 33 | 60 | 10:50 | Euthanasia | 1.26 | 1.37 | 1.0 | 1.0 | 5 | 6 | 0 | A3B3C3 | 1 | 0 |  |  |  |  |  |  |  |  |  |  |  |  |  |  |  |  |  |  |  |
| 39 | PCA | M | 65 | 3 | 3 | 43 | 68 | 6:22 | Dehydration | 1.82 | 1.25 | 3.5 | 2.0 | 5 | 6 | 0 | A3B3C3 | 1 | 0 |  |  |  |  |  |  |  |  |  |  |  |  |  |  |  |  |  |  |  |
| 40 | lvPPA | M | 70 | 5 | 3 | 44 | 75 | 8:35 | Epileptic seizure | 1.65 | 1.24 | 4.0 | 3.0 | 5 | 6 | 0 | A3B3C3 | 3 | 3 |  |  |  |  |  |  |  |  |  |  |  |  |  |  |  |  |  |  |  |
| 41 | lvPPA | F | 66 | 1 | 3 | 33 | 67 | 8:15 | End-stage disease | 1.63 | 1.28 | 2.0 | 1.0 | 5 | 5 | 0 | A3B3C3 | 1 | 0 |  |  |  |  |  |  |  |  |  |  |  |  |  |  |  |  |  |  |  |
| PD | | | | | | | | | | | | | | | | | | | |  |  |  |  |  |  |  |  |  |  |  |  |  |  |  |  |  |  |  |
| 42 | PD | F | 61 | 22 | NA | NA | 83 | 10:35 | Euthanasia | 1.48 | 1.43 | NA | 3.0 | 0 | 1 | 5 | A0B1C0 | 0 | 0 |  |  |  |  |  |  |  |  |  |  |  |  |  |  |  |  |  |  |  |
| 43 | PD | F | 55 | 14 | 0.5 | NA | 69 | 07:05 | Aspiration pneumonia | 1.79 | 1.47 | 3.0 | 1.0 | 2 | 2 | 6 | A1B1C0 | 0 | 0 |  |  |  |  |  |  |  |  |  |  |  |  |  |  |  |  |  |  |  |
| 44 | PD | F | 65 | 17 | NA | NA | 82 | 09:17 | Aspiration pneumonia | 1.52 | 1.43 | 2.0 | 3.0 | 2 | 2 | 6 | A1B1C0 | 0 | 0 |  |  |  |  |  |  |  |  |  |  |  |  |  |  |  |  |  |  |  |
| 45 | PD | M | 61 | 17 | 0.5 | NA | 78 | 07:15 | Euthanasia | 1.46 | 1.43 | 1.0 | 3.0 | 1 | 2 | 6 | A1B1C0 | 0 | 0 |  |  |  |  |  |  |  |  |  |  |  |  |  |  |  |  |  |  |  |
| 46 | PD | M | 77 | 15 | NA | NA | 92 | 10:10 | Myocardial infarction | 1.78 | 1.40 | 2.0 | 3.0 | 3 | 3 | 4 | A2B2C1 | 1 | 0 |  |  |  |  |  |  |  |  |  |  |  |  |  |  |  |  |  |  |  |
| 47 | PD | M | 55 | 20 | 0.5 | NA | 75 | 4:55 | End-stage disease | 1.74 | 1.41 | NA | 3.0 | 3 | 2 | 6 | A2B1C0 | 1 | 0 |  |  |  |  |  |  |  |  |  |  |  |  |  |  |  |  |  |  |  |
| 48 | PD | M | 61 | 17 | NA | NA | 78 | 3:30 | End-stage disease | 1.71 | 1.37 | NA | 1.0 | 1 | 2 | 6 | A1B1C0 | 0 | 0 |  |  |  |  |  |  |  |  |  |  |  |  |  |  |  |  |  |  |  |
| 49 | PD | M | 70 | 23 | NA | NA | 93 | 10:40 | Euthanasia | 1.69 | 1.45 | 1.5 | NA | 2 | 4 | 6 | A1B2C0 | 0 | 0 |  |  |  |  |  |  |  |  |  |  |  |  |  |  |  |  |  |  |  |
| 50 | PD | M | NA | NA | NA | NA | 83 | 6:30 | Euthanasia | 1.75 | 1.39 | 1.0 | 1.0 | 1 | 3 | 6 | A1B2C0 | 0 | 0 |  |  |  |  |  |  |  |  |  |  |  |  |  |  |  |  |  |  |  |
| 51 | PD | M | NA | NA | NA | NA | 84 | 10:30 | Euthanasia | 1.74 | 1.28 | 2.0 | 2.0 | 1 | 3 | 6 | A1B2C0 | 1 | 2 |  |  |  |  |  |  |  |  |  |  |  |  |  |  |  |  |  |  |  |
| 52 | PDD | F | 67 | 16 | 3 | NA | 83 | 10:40 | End-stage disease | 1.53 | 1.41 | 4.0 | 1.0 | 4 | 4 | 6 | A3B2C2 | 1 | 2 |  |  |  |  |  |  |  |  |  |  |  |  |  |  |  |  |  |  |  |
| 53 | PDD | F | 84 | 10 | 3 | NA | 94 | 06:50 | Palliative sedation | 1.48 | 1.39 | 1.0 | 2.0 | 4 | 4 | 6 | A3B2C2 | 0 | 2 |  |  |  |  |  |  |  |  |  |  |  |  |  |  |  |  |  |  |  |
| 54 | PDD | F | 62 | 12 | 1 | NA | 74 | 08:10 | Euthanasia | 1.46 | 1.35 | 2.5 | 1.0 | 3 | 2 | 6 | A2B1C0 | 1 | 0 |  |  |  |  |  |  |  |  |  |  |  |  |  |  |  |  |  |  |  |
| 55 | PDD | M | 58 | 21 | 2 | NA | 79 | 09:25 | Subarachnoid bleeding | 1.94 | 1.33 | 1.0 | 2.0 | 3 | 2 | 6 | A2B1C1 | 1 | 0 |  |  |  |  |  |  |  |  |  |  |  |  |  |  |  |  |  |  |  |
| 56 | PDD | M | 44 | 18 | NA | NA | 62 | 05:10 | End-stage disease | 1.47 | 1.47 | 1.0 | 2.0 | 1 | 2 | 6 | A1B1C0 | 0 | 0 |  |  |  |  |  |  |  |  |  |  |  |  |  |  |  |  |  |  |  |
| 57 | PDD | M | 66 | 8 | 2 | NA | 74 | 8:50 | Aspiration pneumonia | 1.85 | 1.27 | 1.0 | 1.0 | 2 | 3 | 6 | A1B2C0 | 0 | 0 |  |  |  |  |  |  |  |  |  |  |  |  |  |  |  |  |  |  |  |
| 58 | PDD | F | 61 | 20 | NA | NA | 81 | 5:30 | End-stage disease | 1.74 | 1.38 | 1.0 | 0.0 | 3 | 2 | 6 | A2B1C0 | 1 | 0 |  |  |  |  |  |  |  |  |  |  |  |  |  |  |  |  |  |  |  |
| 59 | PDD | M | 62 | 8 | 1 | NA | 70 | 6:55 | Euthanasia | 1.55 | 1.51 | 1.5 | 2.0 | 1 | 1 | 6 | A1B1C0 | 0 | 0 |  |  |  |  |  |  |  |  |  |  |  |  |  |  |  |  |  |  |  |
| 60 | PDD | F | NA | NA | NA | NA | 74 | 9:30 | Euthanasia | 1.66 | 1.51 | 1.0 | 1.0 | 1 | 1 | 6 | A1B1C0 | 0 | 0 |  |  |  |  |  |  |  |  |  |  |  |  |  |  |  |  |  |  |  |

**Legend:**

*AD: Alzheimer’s disease; B/D: behavioral/dysexecutive; CAA: cerebral amyloid angiopathy; CDR: clinical dementia rating; CTRL: control; F: female; HP: hippocampus; ICV: intracranial volume; LATE: limbic-predominant age-related TDP-43 encephalopathy; LB:Lewy bodies; lvPPA; logopenic variant primary progressive aphasia; M: male; MTA: medial temporal lobe atrophy; NA: not applicable; NFT: neurofibrillary tangles; PCA: posterior cortical atrophy; PD: Parkinson’s disease; PDD: Parkinson’s disease dementia; PMD: post-mortem delay.*

**Supplementary Table 2.** Information on primary antibodies

| Primary antibody | Antigen | Species | Company | Dilution | Incubation | Antigen retrieval | Detection method |
| --- | --- | --- | --- | --- | --- | --- | --- |
| amyloid-β, clone 4G8 | Aβ amino acid sequence 17-24 | Mouse igG2b | BioLegend, San Diego. USA | 1:8000 | 4°C overnight | Citrate buffer (pH 6.0) in steam cooker | EnVision (HRP) |
| p-tau. clone AT8 | Tau phosphorylated at Ser202 and Thr205 | Mouse igG1 | ThermoFisher, Pittsburgh. USA | 1:800 | 4°C overnight | Citrate buffer (pH 6.0) in steam cooker | EnVision (HRP) |
| pSer129 aSyn, clone EP1536Y | Alpha synuclein phosphorylated at Ser129 | Rabbit igG | Abcam. Cambridge, UK | 1:8000 | 4°C overnight | Tris EDTA buffer (pH 9.0) in steam cooker | EnVision (HRP) |

**Supplementary Table 3.** R-values and p-values before and after FDR-correction of MRI volume-pathology correlations in controls. As none of the uncorrected p-values were significant. FDR-correction was not possible.

|  |  | **r-value** | **uncorrected p-value** | **FDR-corrected p-value** |
| --- | --- | --- | --- | --- |
| **amyloid-β** | Total hippocampus | 0.08 | 0.900 | - |
|  | DG | 0.49 | 0.193 | - |
|  | CA4 | 0.47 | 0.226 | - |
|  | CA2/3 | 0.33 | 0.390 | - |
|  | CA1 | 0.36 | 0.530 | - |
|  | Subiculum | -0.01 | 0.537 | - |
|  | Parasubiculum | -0.05 | 0.326 | - |
|  | Entorhinal cortex | -0.33 | 0.549 | - |
| **p-tau** | Total hippocampus | -0.48 | 0.305 | - |
|  | DG | 0.31 | 0.378 | - |
|  | CA4 | 0.37 | 0.304 | - |
|  | CA2/3 | 0.62 | 0.104 | - |
|  | CA1 | -0.37 | 0.328 | - |
|  | Subiculum | -0.33 | 0.465 | - |
|  | Parasubiculum | -0.02 | 0.955 | - |
|  | Entorhinal cortex | 0.09 | 0.758 | - |

**Supplementary Table 4.** R-values and p-values before and after FDR-correction of MRI volume-pathology correlations in AD. As none of the uncorrected p-values were significant. FDR-correction was not possible.

|  |  | **r-value** | **uncorrected p-value** | **FDR-corrected p-value** |
| --- | --- | --- | --- | --- |
| **amyloid-β** | Total hippocampus | 0.21 | 0.334 | - |
|  | DG | 0.34 | 0.111 | - |
|  | CA4 | 0.27 | 0.219 | - |
|  | CA2/3 | 0.19 | 0.391 | - |
|  | CA1 | 0.35 | 0.104 | - |
|  | Subiculum | 0.02 | 0.927 | - |
|  | Parasubiculum | -0.15 | 0.486 | - |
|  | Entorhinal cortex | -0.01 | 0.983 | - |
| **p-tau** | Total hippocampus | -0.09 | 0.690 | - |
|  | DG | -0.04 | 0.845 | - |
|  | CA4 | -0.17 | 0.432 | - |
|  | CA2/3 | -0.05 | 0.817 | - |
|  | CA1 | -0.14 | 0.516 | - |
|  | Subiculum | -0.25 | 0.258 | - |
|  | Parasubiculum | -0.14 | 0.526 | - |
|  | Entorhinal cortex | -0.05 | 0.832 | - |

**Supplementary Table 5.** R-values and p-values before and after FDR-correction of MRI volume-pathology correlations in typical and atypical AD. As none of the uncorrected p-values were significant. FDR-correction was not possible.

| **Typical AD** | | | | |
| --- | --- | --- | --- | --- |
|  |  | **r-value** | **uncorrected p-value** | **FDR-corrected p-value** |
| **amyloid-β** | Total hippocampus | -0.03 | 0.943 | - |
|  | DG | 0.27 | 0.457 | - |
|  | CA4 | 0.31 | 0.385 | - |
|  | CA2/3 | -0.04 | 0.908 | - |
|  | CA1 | 0.06 | 0.877 | - |
|  | Subiculum | -0.10 | 0.786 | - |
|  | Parasubiculum | -0.49 | 0.155 | - |
|  | Entorhinal cortex | -0.23 | 0.516 | - |
| **p-tau** | Total hippocampus | -0.27 | 0.452 | - |
|  | DG | 0.10 | 0.788 | - |
|  | CA4 | -0.03 | 0.925 | - |
|  | CA2/3 | -0.11 | 0.771 | - |
|  | CA1 | -0.38 | 0.276 | - |
|  | Subiculum | -0.30 | 0.405 | - |
|  | Parasubiculum | -0.37 | 0.300 | - |
|  | Entorhinal cortex | -0.31 | 0.391 | - |
| **Atypical AD** | | | | |
|  |  | **r-value** | **uncorrected p-value** | **FDR-corrected p-value** |
| **amyloid-β** | Total hippocampus | 0.54 | 0.135 | - |
|  | DG | 0.52 | 0.149 | - |
|  | CA4 | 0.69 | 0.038 | - |
|  | CA2/3 | 0.69 | 0.038 | - |
|  | CA1 | 0.34 | 0.368 | - |
|  | Subiculum | 0.25 | 0.509 | - |
|  | Parasubiculum | 0.42 | 0.257 | - |
|  | Entorhinal cortex | 0.26 | 0.492 | - |
| **p-tau** | Total hippocampus | 0.73 | 0.024 | - |
|  | DG | 0.52 | 0.153 | - |
|  | CA4 | 0.48 | 0.190 | - |
|  | CA2/3 | 0.40 | 0.292 | - |
|  | CA1 | 0.60 | 0.087 | - |
|  | Subiculum | 0.20 | 0.612 | - |
|  | Parasubiculum | 0.45 | 0.225 | - |
|  | Entorhinal cortex | 0.06 | 0.877 | - |

**Supplementary Table 6.** R-values and p-values before and after FDR-correction of MRI volume-pathology correlations in PD. As none of the uncorrected p-values were significant for pSer129-αSyn and amyloid- β. FDR-correction was not possible for these pathological markers.

|  |  | **r-value** | **uncorrected p-value** | **FDR-corrected p-value** |
| --- | --- | --- | --- | --- |
| **pSer129-αSyn** | Total hippocampus | -0.54 | 0.046 | - |
|  | DG | -0.44 | 0.115 | - |
|  | CA4 | -0.51 | 0.063 | - |
|  | CA2/3 | -0.34 | 0.232 | - |
|  | CA1 | -0.29 | 0.310 | - |
|  | Subiculum | -0.48 | 0.098 | - |
|  | Parasubiculum | -0.44 | 0.120 | - |
|  | Entorhinal cortex | -0.23 | 0.416 | - |
| **p-tau** | **Total hippocampus** | **-0.68** | **0.006** | **0.045** |
|  | DG | -0.41 | 0.133 | 1.000 |
|  | CA4 | -0.50 | 0.056 | 0.452 |
|  | CA2/3 | -0.41 | 0.125 | 1.000 |
|  | CA1 | -0.34 | 0.216 | 1.000 |
|  | Subiculum | -0.55 | 0.035 | 0.282 |
|  | Parasubiculum | -0.45 | 0.090 | 0.722 |
|  | Entorhinal cortex | -0.15 | 0.597 | 1.000 |
| **amyloid-β** | Total hippocampus | -0.35 | 0.204 | - |
|  | DG | -0.21 | 0.466 | - |
|  | CA4 | -0.12 | 0.674 | - |
|  | CA2/3 | -0.27 | 0.390 | - |
|  | CA1 | 0.02 | 0.530 | - |
|  | Subiculum | -0.23 | 0.537 | - |
|  | Parasubiculum | -0.47 | 0.326 | - |
|  | Entorhinal cortex | -0.01 | 0.549 | - |

**Supplementary Table 7.** R-values and p-values before and after FDR-correction of MRI volume-pathology correlations in PD and PDD subgroups. As none of the uncorrected p-values were significant in PD and for pSer129-αSyn and amyloid- β in PDD. FDR-correction was not possible for these pathological markers.

| **PD** | | | | |
| --- | --- | --- | --- | --- |
|  |  | **r-value** | **uncorrected p-value** | **FDR-corrected p-value** |
| **pSer129-αSyn** | Total hippocampus | -0.01 | 0.990 | - |
|  | DG | -0.78 | 0.121 | - |
|  | CA4 | -0.38 | 0.534 | - |
|  | CA2/3 | -0.44 | 0.455 | - |
|  | CA1 | 0.42 | 0.486 | - |
|  | Subiculum | -0.73 | 0.271 | - |
|  | Parasubiculum | -0.63 | 0.252 | - |
|  | Entorhinal cortex | 0.13 | 0.801 | - |
| **p-tau** | Total hippocampus | 0.16 | 0.758 | - |
|  | DG | 0.05 | 0.923 | - |
|  | CA4 | -0.10 | 0.845 | - |
|  | CA2/3 | 0.10 | 0.845 | - |
|  | CA1 | 0.10 | 0.845 | - |
|  | Subiculum | -0.16 | 0.842 | - |
|  | Parasubiculum | 0.35 | 0.494 | - |
|  | Entorhinal cortex | 0.28 | 0.586 | - |
| **amyloid-β** | Total hippocampus | 0.48 | 0.339 | - |
|  | DG | 0.62 | 0.187 | - |
|  | CA4 | 0.67 | 0.149 | - |
|  | CA2/3 | 0.51 | 0.306 | - |
|  | CA1 | 0.54 | 0.266 | - |
|  | Subiculum | 0.48 | 0.330 | - |
|  | Parasubiculum | -0.28 | 0.598 | - |
|  | Entorhinal cortex | 0.51 | 0.301 | - |
| **PDD** | | | | |
|  |  | **r-value** | **uncorrected p-value** | **FDR-corrected p-value** |
| **pSer129-αSyn** | Total hippocampus | -0.72 | 0.170 | - |
|  | DG | -0.51 | 0.375 | - |
|  | CA4 | -0.75 | 0.148 | - |
|  | CA2/3 | -0.36 | 0.547 | - |
|  | CA1 | -0.38 | 0.527 | - |
|  | Subiculum | -0.57 | 0.320 | - |
|  | Parasubiculum | -0.54 | 0.350 | - |
|  | Entorhinal cortex | -0.35 | 0.560 | - |
| **p-tau** | **Total hippocampus** | **-0.99** | **0.002** | **0.013** |
|  | DG | -0.92 | 0.025 | 0.201 |
|  | CA4 | -0.91 | 0.031 | 0.251 |
|  | CA2/3 | -0.49 | 0.399 | 1.000 |
|  | CA1 | -0.42 | 0.478 | 1.000 |
|  | Subiculum | -0.77 | 0.127 | 1.000 |
|  | Parasubiculum | -0.87 | 0.056 | 0.449 |
|  | Entorhinal cortex | -0.52 | 0.368 | 1.000 |
| **amyloid-β** | Total hippocampus | -0.66 | 0.221 | - |
|  | DG | -0.62 | 0.261 | - |
|  | CA4 | -0.51 | 0.384 | - |
|  | CA2/3 | 0.14 | 0.826 | - |
|  | CA1 | -0.10 | 0.873 | - |
|  | Subiculum | -0.61 | 0.274 | - |
|  | Parasubiculum | -0.73 | 0.163 | - |
|  | Entorhinal cortex | -0.31 | 0.690 | - |

# References

1. Thal DR. Rub U. Orantes M. Braak H. Phases of A beta-deposition in the human brain and its relevance for the development of AD. Neurology. 2002;58(12):1791-800.

2. Braak H. Alafuzoff I. Arzberger T. Kretzschmar H. Del Tredici K. Staging of Alzheimer disease-associated neurofibrillary pathology using paraffin sections and immunocytochemistry. Acta Neuropathol. 2006;112(4):389-404.

3. Braak H. Del Tredici K. Rub U. de Vos RA. Jansen Steur EN. Braak E. Staging of brain pathology related to sporadic Parkinson's disease. Neurobiol Aging. 2003;24(2):197-211.

4. Montine TJ. Phelps CH. Beach TG. Bigio EH. Cairns NJ. Dickson DW. et al. National Institute on Aging-Alzheimer's Association guidelines for the neuropathologic assessment of Alzheimer's disease: a practical approach. Acta Neuropathol. 2012;123(1):1-11.
