# Supplementary material for: Hippocampal subfields: volume, neuropathological vulnerability and cognitive decline in Alzheimer’s and Parkinson’s disease
Source: Alzheimers Res Ther. 2025 May 30;17:121. doi: 10.1186/s13195-025-01768-w (PMC12124080; doi:10.1186/s13195-025-01768-w)
Supplement: Supplementary file 2 — Supplementary Material 2 [file 13195_2025_1768_MOESM2_ESM.docx]

**
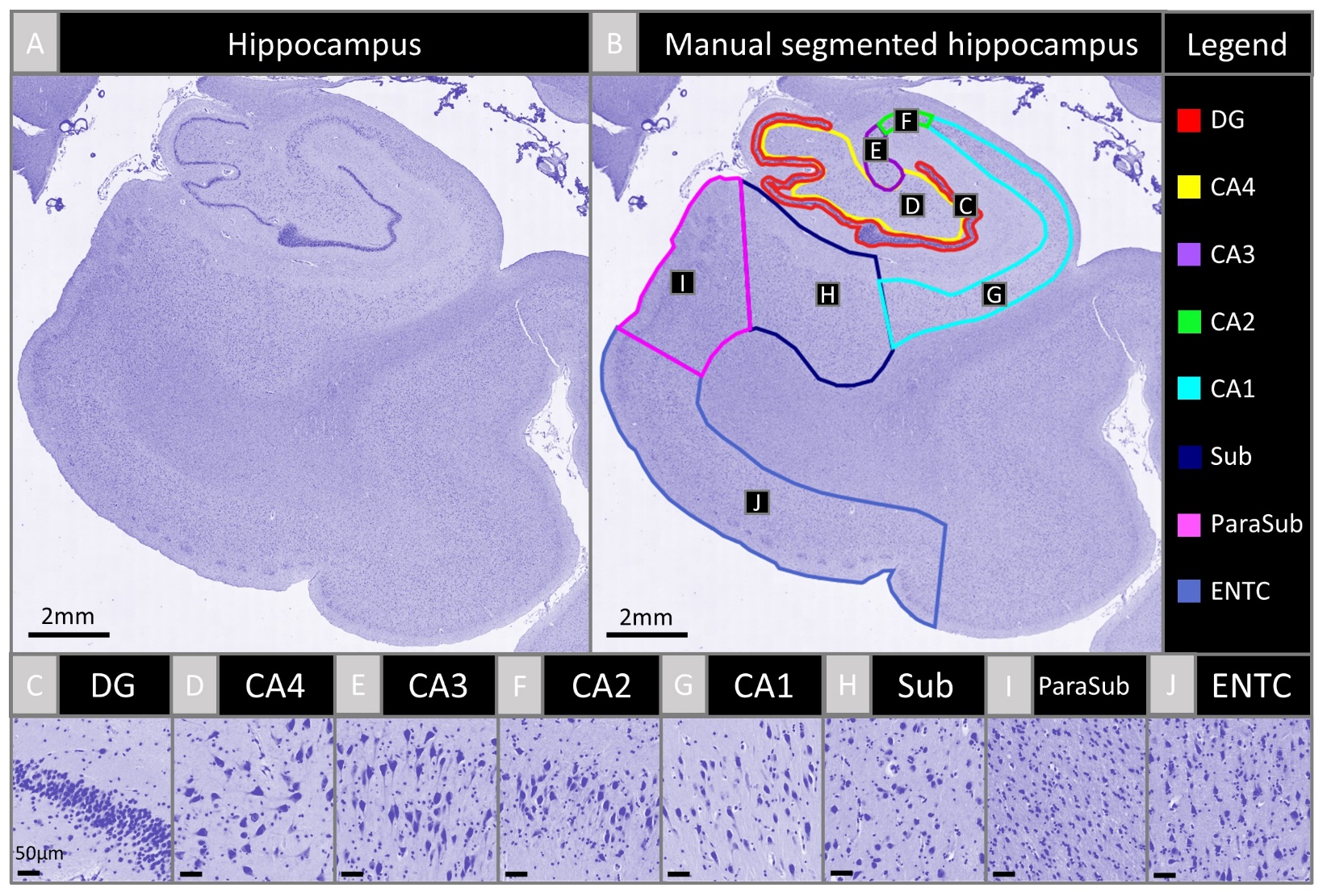
Supplementary figures**

**Suppl. Fig. 1. Manual segmented hippocampal subfields in QuPath.** Hippocampal section depicted with a counterstaining of hematoxylin (**A**) and manually segmented into different subfields (**B**), according to the cytoarchitecture as described previously^49^, which is shown per subfield on the bottom (**C-J**; corresponding to the black boxes in **B**). Briefly, the DG was defined by small, round and densely packed granular neurons with the hilar region as CA4. The somata of the CA3 appeared similar to those in CA4 but more triangular and dense. CA2 pyramidal layer was the thinnest of all CA subfields and the CA3/CA2 transition was characterized by more densely packed pyramidal neurons. By contrast, CA1 neuronal somata were more ovoid shaped and more sparsely populated. The pyramidal cells in the subiculum were similar to CA1, but with a more striated appearance. The subiculum consisted of three cellular layers. The parasubiculum was started where six layers were recognizable, with granular islands in layer II, and transitioned into ENTC where the layering pattern became more clear, especially with layer IV becoming lamina dissecans. The ENTC ended where layer IV of the parahippocampal gyrus started to be visible. **Legend**: *CA: cornu ammonis; DG: dentate gyrus; ENTC: entorhinal cortex; ParaSub: parasubiculum; Sub: subiculum.*

**
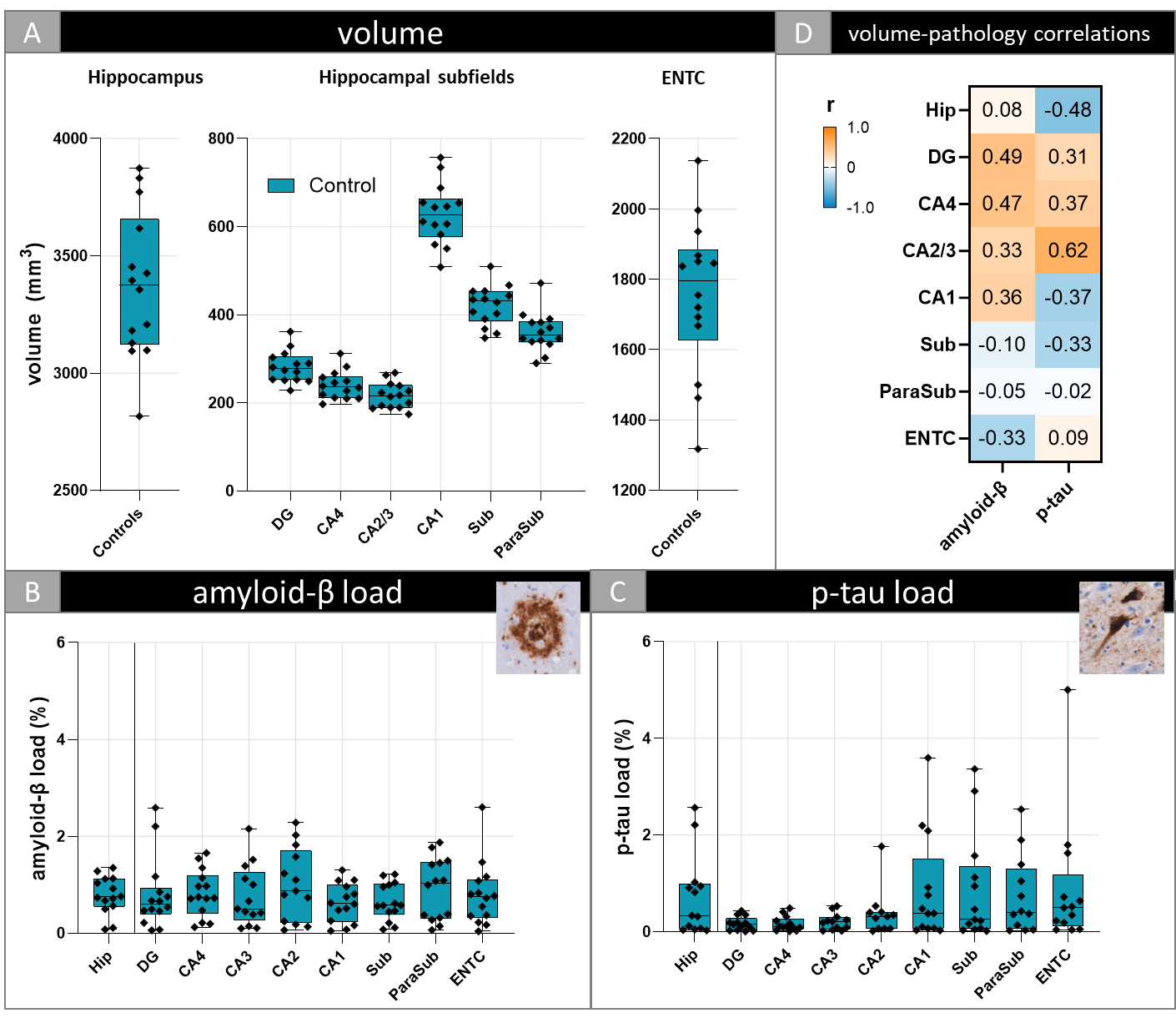
Suppl. Fig. 2. Hippocampal subfield MRI volumes, amyloid-β and p-tau load in controls.** MRI volumes (**A**), amyloid-β (**B**) and p-tau load (**C**) of the different hippocampal subfields in non-neurological controls. The boxes indicate the median with 25^th^ and 75^th^ percentile. The correlations between volume and pathology load are shown in the heatmap (**D**), color-coded for correlation coefficient (r): blue represents negative and orange positive correlation. No significant correlations were found (all p>0.05). **Legend:** *CA: Cornu Ammonis; CTRL: controls; DG: dentate gyrus; Hip: total hippocampus; ENTC: entorhinal cortex; ParaSub: parasubiculum; Sub: subiculum.*

**
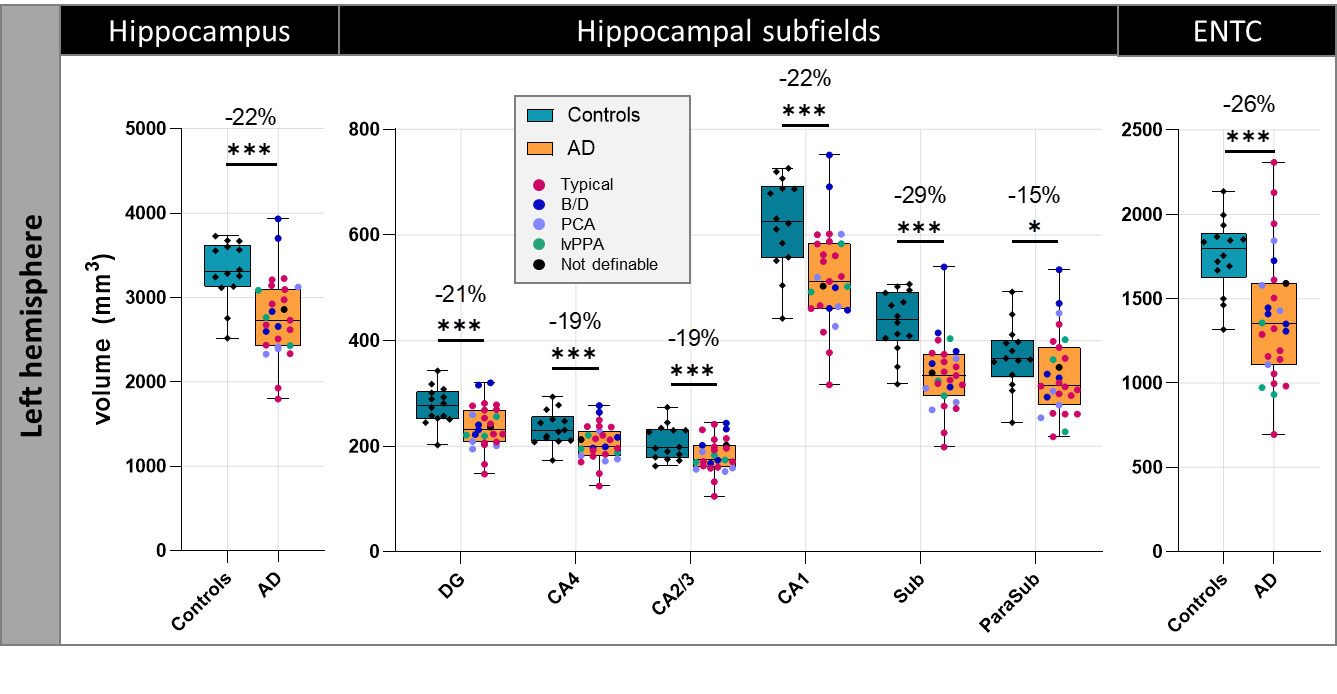
**

**Suppl. Fig. 3. Left hemisphere hippocampal subfield volume differences between controls and AD.** Volumetric differences between controls and AD donors from the left hemisphere are shown with every data point representing one donor and color-coded based on clinical phenotype. The boxes indicate the median with 25^th^ and 75^th^ percentile. * p<0.05, ** p<0.01, *** p<0.001 The percentages depicted are the percentages of difference in estimated marginal means, taking into account the influence of covariates. These volumetric measurements were not used for further correlation analysis due to contralateral pathological data. **Legend:** *AD: Alzheimer’s disease; B/D: behavioral/dysexecutive; CA: Cornu Ammonis; DG: dentate gyrus; ENTC: entorhinal cortex; lvPPA; logopenic variant primary progressive aphasia; ParaSub: parasubiculum; PCA: posterior cortical atrophy;* *Sub: subiculum.*

**
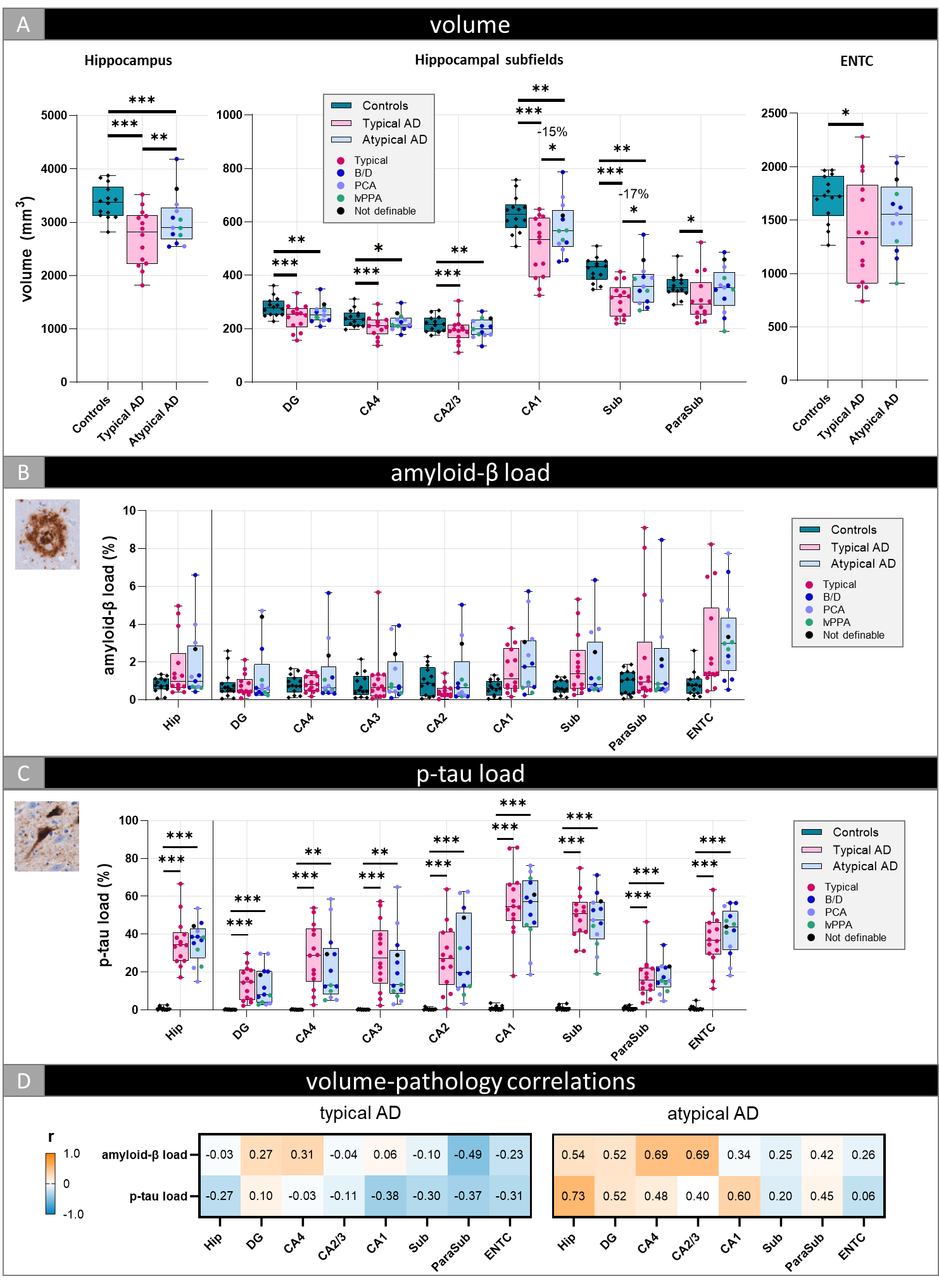
**

**Suppl. Fig. 4. Hippocampal subfield volume and pathology differences between controls and typical and atypical AD.** Volumetric (**A**) and amyloid β (**B**) and p-tau (**C**) load differences between controls and typical and atypical AD donors are shown for the total hippocampus and per hippocampal subregion and correlations between volume and pathology load for the typical and atypical AD group (**D**). Every data point represents one donor and color-coded based on clinical phenotype. The boxes indicate the median with 25^th^ and 75^th^ percentile. All p-values were FDR-corrected for multiple comparisons. * p<0.05, ** p<0.01, *** p<0.001. The percentages depicted in (**A**) is the percentage of difference in estimated marginal means, taking into account the influence of covariates. The heatmaps in (**D**) are color-coded for correlation coefficient (r): blue represents negative and orange positive correlations and the numbers in the heatmaps represent the p-values corresponding with the correlation. **Legend:** *AD: Alzheimer’s disease; B/D: behavioral/dysexecutive; CA: Cornu Ammonis; DG: dentate gyrus; ENTC: entorhinal cortex; lvPPA; logopenic variant primary progressive aphasia; ParaSub: parasubiculum; PCA: posterior cortical atrophy;* *Sub: subiculum.*

**
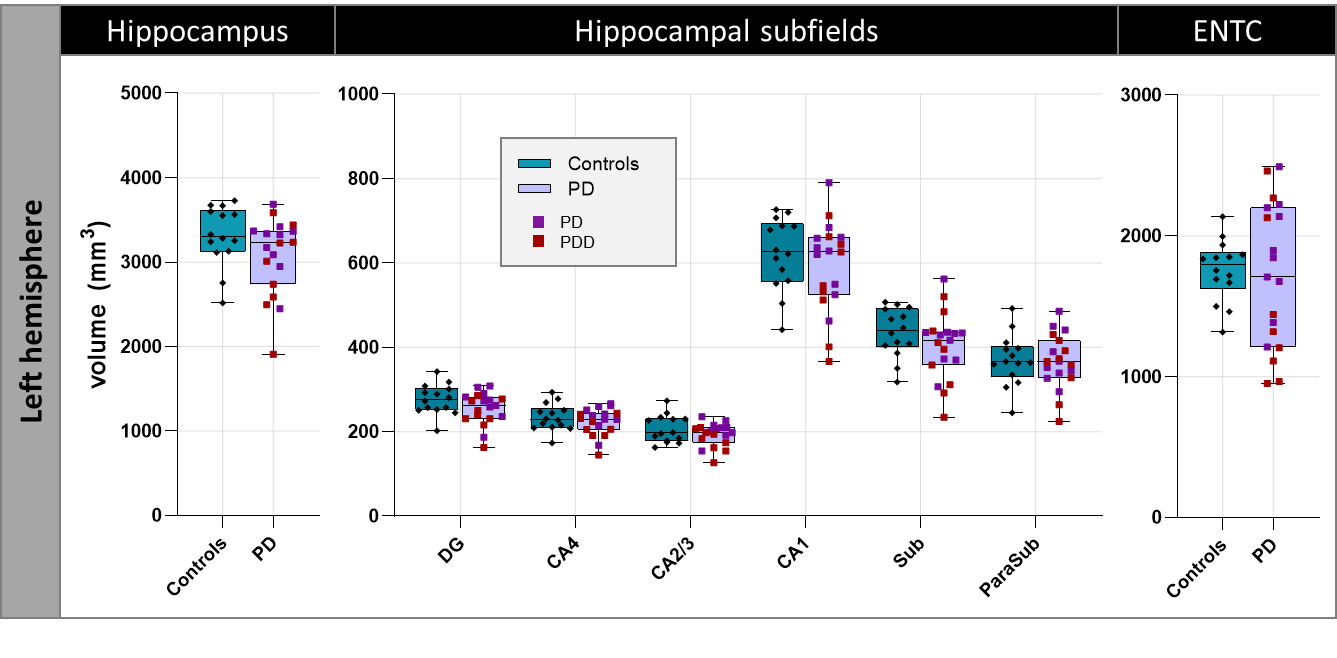
Suppl. Fig. 5. Left hemisphere hippocampal subfield volume differences between controls and PD.** Volumetric differences between controls and PD donors from the left hemisphere are shown with every data point representing one donor and color-coded based on the presence of dementia (PD vs. PDD). These volumetric measurements were not used for further correlation analysis due to contralateral pathological data. The boxes indicate the median with 25^th^ and 75^th^ percentile. **Legend:** *CA: Cornu Ammonis; DG: dentate gyrus; ENTC: entorhinal cortex; ParaSub: parasubiculum;* *PD: Parkinson’s disease; PDD: Parkinson’s disease dementia; Sub: subiculum.*

**
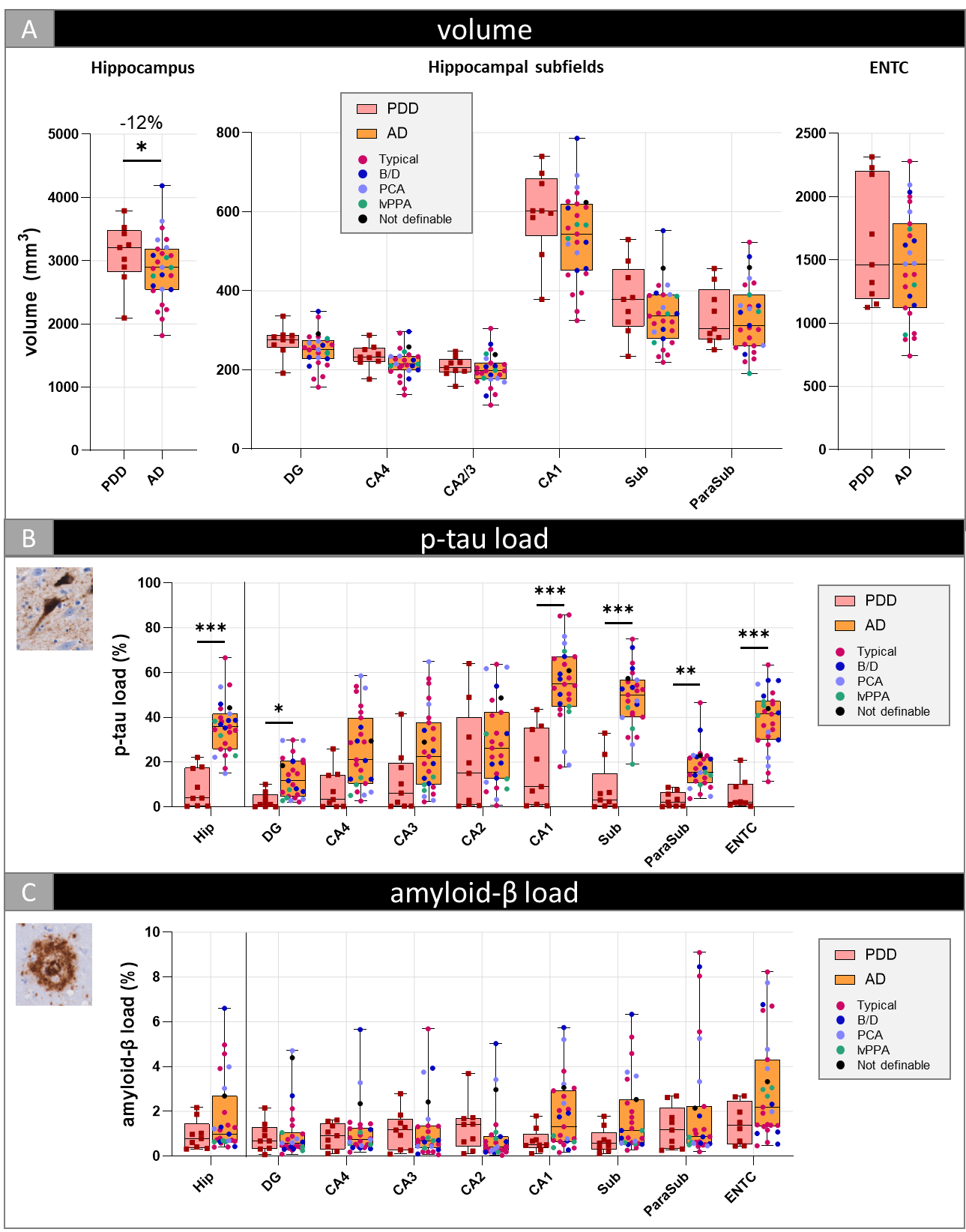
**

**Suppl. Fig. 6. Hippocampal subfield pathology load differences between PDD and AD.** Volumetric (A), p-tau (**B**) and amyloid β load (**C**) differences between PDD and AD are shown for the total hippocampus and per hippocampal subregion. Every data point represents one donor, and for the AD cohort color-coded based on clinical phenotype. The boxes indicate the median with 25^th^ and 75^th^ percentile. * p<0.05, ** p<0.01, *** p<0.001 The percentage depicted in (A) is the percentage of difference in estimated marginal means, taking into account the influence of covariates. **Legend:** *AD: Alzheimer’s disease; B/D: behavioral/dysexecutive; CA: Cornu Ammonis; DG: dentate gyrus; ENTC: entorhinal cortex; Hip: total hippocampus; lvPPA; logopenic variant primary progressive aphasia; ParaSub: parasubiculum; PCA: posterior cortical atrophy;* *PDD: Parkinson’s disease dementia;Sub: subiculum.*
